# Optical and Electrochemical Biosensors for Detection of Pathogens Using Metal Nanoclusters: A Systematic Review

**DOI:** 10.3390/bios15070460

**Published:** 2025-07-17

**Authors:** Mahsa Shahrashoob, Mahdiyar Dehshiri, Vahid Yousefi, Mahdi Moassesfar, Hamidreza Saberi, Fatemeh Molaabasi, Yasser Zare, Kyong Yop Rhee

**Affiliations:** 1 Department of Biochemistry and Biophysics, TeMS.C., Islamic Azad University, Tehran 1811694784, Iran; m.shahrashoob@iau.ir (M.S.); mahdi.moassesfar@iau.ir (M.M.); 2 Medical Nanotechnology Group, Department of Interdisciplinary Technologies, Breast Cancer Research Center, Motamed Cancer Institute, ACECR, Tehran 1517964311, Iran; m.dehshiri@modares.ac.ir (M.D.); vahid.usefi89@gmail.com (V.Y.); hrsaberi@ut.ac.ir (H.S.); 3 Department of Nanobiotechnology, Faculty of Biological Sciences, Tarbiat Modares University, Tehran 1458889694, Iran; 4 Biomaterials and Tissue Engineering Research Group, Department of Interdisciplinary Technologies, Breast Cancer Research Center, Motamed Cancer Institute, ACECR, Tehran 1517964311, Iran; y.zare@aut.ac.ir; 5 Department of Mechanical Engineering (BK21 Four), College of Engineering, Kyung Hee University, Yongin 17104, Republic of Korea

**Keywords:** metal nanocluster-based biosensors, virus detection, bacterial detection, microbial infections, nanobiosensors

## Abstract

The rapid and accurate detection of pathogenic bacteria and viruses is critical for infectious disease control and public health protection. While conventional methods (e.g., culture, microscopy, serology, and PCR) are widely used, they are often limited by lengthy processing times, high costs, and specialized equipment requirements. In recent years, metal nanocluster (MNC)-based biosensors have emerged as powerful diagnostic platforms due to their unique optical, catalytic, and electrochemical properties. This systematic review comprehensively surveys advancements in MNC-based biosensors for bacterial and viral pathogen detection, focusing on optical (colorimetric and fluorescence) and electrochemical platforms. Three key aspects are emphasized: (1) detection mechanisms, (2) nanocluster types and properties, and (3) applications in clinical diagnostics, environmental monitoring, and food safety. The literature demonstrates that MNC-based biosensors provide high sensitivity, specificity, portability, and cost-efficiency. Moreover, the integration of nanotechnology with biosensing platforms enables real-time and point-of-care diagnostics. This review also discusses the limitations and future directions of the technology, emphasizing the need for enhanced stability, multiplex detection capability, and clinical validation. The findings offer valuable insights for developing next-generation biosensors with improved functionality and broader applicability in microbial diagnostics.

## 1. Introduction

The rapid detection of pathogenic bacteria and viruses is fundamental to safeguarding public health, enabling timely and effective medical interventions, preventing the spread of infectious diseases, and ensuring environmental safety [1,2,3]. Infectious diseases can significantly impact the economy and impose substantial pressure on healthcare systems. During the COVID-19 pandemic, significant financial resources were directed toward vaccination programs and medical research. these outbreaks could disrupt daily life, force businesses to shut down, and lead to significant economic losses [4,5].

Traditional methods for detecting bacteria and viruses rely on culture techniques [6,7], microscopy [8,9], and serological assays [10,11]. Molecular diagnostic techniques, such as PCR, are also widely used to detect microbial infections [12,13,14]. However, these methods can be laborious and have several limitations, including time-consuming processes, high costs, dependence on specialized equipment, and the need for skilled personnel [15,16,17,18]. Therefore, there is a need for alternative approaches that employ simple, rapid, sensitive, and cost-effective methods to detect bacteria and viruses. For example, many papers propose molecular imprinted polymers (MIPs) for this purpose [19]. Biosensors offer a promising alternative for detecting microbial infections, addressing the limitations of traditional diagnostic methods [20,21].

Biosensors are analytical tools that incorporate a biological recognition element and convert a biological or chemical response into a signal proportional to the analyte’s concentration. The analyte’s interaction with the bioreceptor produces an effect detected by the transducer, which converts it into a measurable signal, such as an electrical or optical signal [22,23]. Nanomaterials possessing advantageous properties have significantly advanced this field, particularly by enhancing sensitive and rapid detection [24].

Among various nanomaterials, metal nanoclusters (MNCs) have drawn considerable interest for biosensing applications. MNCs exhibit strong photoluminescence, high photochemical stability, catalytic activity, good water solubility, ease of synthesis, and biocompatibility. Consequently, MNCs serve as highly effective biosensor candidates, enhancing sensitivity, selectivity, and detection efficiency [25,26,27,28]. These nanoclusters differ significantly from traditional nanoparticles (NPs) due to their ultra-small size, which often leads to distinctive optical behavior compared to larger NPs [29,30,31]. Silver nanoclusters (AgNCs), gold nanoclusters (AuNCs), and copper nanoclusters (CuNCs) have attracted significant attention in biosensor development due to their broad applicability and simple synthesis. They allow precise detection at low detection limits, making them suitable for these applications [32,33,34,35]. MNCs can be synthesized using various methods, including etching techniques [36], chemical reduction [37], template-assisted synthesis [38,39], monolayer-protected methods [40], irradiation-based approaches [41], photoreduction [42], inert-gas condensation [43], ultrasonic synthesis [44], and other related techniques. Generally, these methods are classified into two main synthetic routes for MNCs: bottom-up (the more common approach, involving the assembly of nanoclusters from smaller precursors) or top-down (etching larger nanomaterials into smaller nanostructures) [33,45]. Recently, interest has grown in using MNC-based biosensors to detect viral and bacterial pathogens, especially in medical diagnostics, environmental monitoring, and food safety analysis [46,47,48]. However, despite the growing body of research on MNC-based biosensors and their promising applications in detecting viral and bacterial pathogens, comprehensive review articles on this topic are still limited. This shortage reflects a clear gap in the literature, emphasizing the need for an in-depth review to consolidate current findings and support further research.

**Scheme 1:**
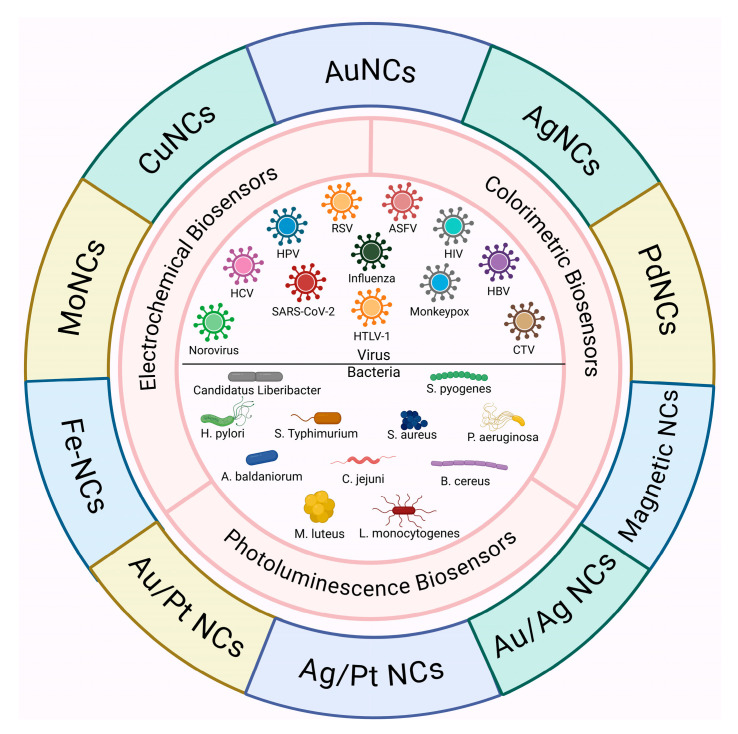
Schematic representation of MNCs applications for photoluminescence, electrochemical, and colorimetric detection of viral and bacterial infections. Created with BioRender.com.

This systematic review surveys advancements in MNC-based biosensors for detecting viral and bacterial infections, focusing on applications in fluorescence, electrochemical, and colorimetric assays. Furthermore, key challenges and future research directions are briefly discussed. The findings of this review are expected to facilitate the development of MNC-based biosensors with enhanced functionalities for detecting bacteria and viruses, addressing the critical need for rapid and sensitive pathogen detection to safeguard public health and economic stability (Scheme 1).

## 2. Methodology

This systematic review was conducted in accordance with the Preferred Reporting Items for Systematic Reviews and Meta-Analyses (PRISMA) guidelines [49].

### 2.1. Literature Research Strategy

A comprehensive literature review was carried out using several online databases, including PubMed, Google Scholar, Scopus, and Web of Science, to cover studies published between January 2007 and February 2025. To ensure broad coverage, we utilized a variety of keywords related to the topic. Our primary search terms included (“clusters” OR “metal nanoclusters”), AND (“biosensors” OR “nanobiosensors”), AND (“bacteria” OR “virus” OR “pathogen”), AND (“optical” OR “colorimetric” OR “fluorescent” OR “electrochemical”), OR “diagnostic applications”, OR “pathogen detection”, OR “point-of-care testing”. Additionally, we incorporated supplementary keywords, including (“gold nanoclusters” OR “silver nanoclusters” OR “copper nanoclusters” OR “nanomaterials”), AND “nanobiosensors “, AND (“HIV” OR “HBV” OR “SARS-CoV-2” OR “HPV” OR “bacteria”) to refine our search results.

### 2.2. Study Selection Process

**Figure 1:**
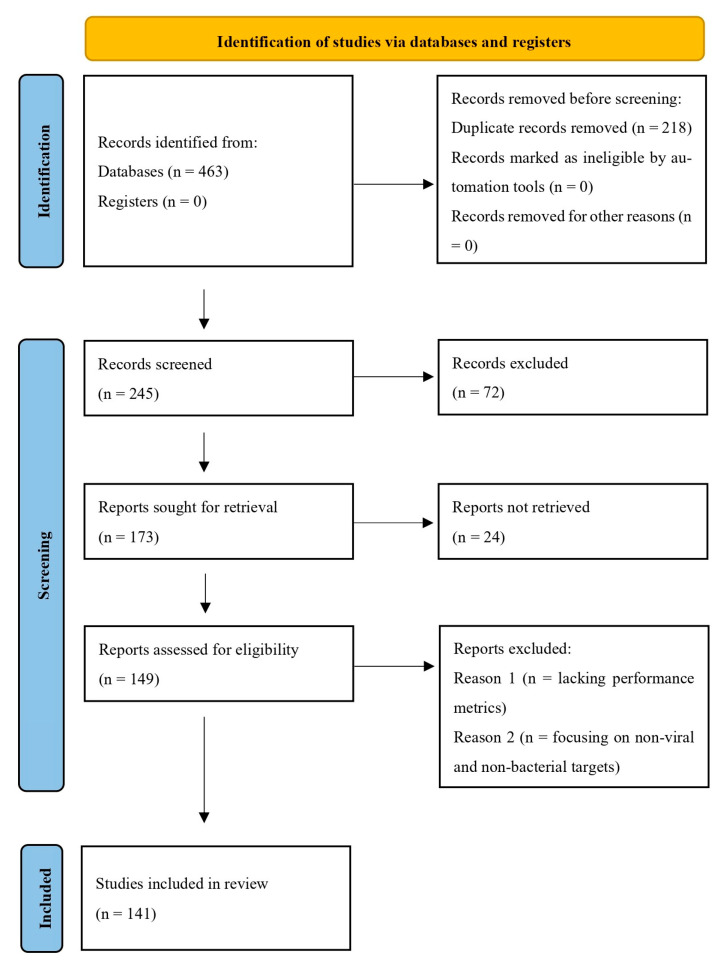
PRISMA flowchart of the search strategy covering the period 2007–2025.

Our initial database search yielded 463 potentially relevant studies. After eliminating 218 duplicate entries, the remaining 245 studies were screened based on their titles and abstracts according to our predefined eligibility criteria. The inclusion criteria for this review encompassed studies that investigated the use of MNC-based biosensors for detecting viruses and bacteria. Only English original research and review articles published in peer-reviewed journals were considered. Studies that included experimental validation of MNC-based biosensors and their applications in virus or bacterial detection were prioritized. Eligible studies provided key performance metrics such as sensitivity, specificity, and the limit of detection (LOD). Furthermore, studies that discussed the clinical or technological aspects of biosensor applications, including fluorescent, calorimetric, and electrochemical detection mechanisms, were included. We excluded case reports, conference abstracts, and non-peer-reviewed articles. Studies written in languages other than English were also excluded to ensure accessibility and the consistency of data interpretation. Studies with incomplete or inconclusive data, those lacking performance metrics, those focusing on non-viral and non-bacterial targets, or those with an indirect address for the use of nanoclusters (NCs) in biosensing applications were not included in the final selection. After applying these criteria, a total of 141 studies were included in our study (Figure 1).

### 2.3. Focus Questions

The focus questions were formulated based on the problem, intervention, comparison, and outcome (PICO) method. The research questions were as follows: What types of MNC-based biosensors could be effective for the rapid detection of viral and bacterial infections? Which MNCs are most commonly used for detecting viral and bacterial pathogens in biosensing applications? Which MNC-based biosensors demonstrate the highest sensitivity and good selectivity in pathogen detection? What types of biological samples have been analyzed using MNC-based biosensors for viral and bacterial detection?

### 2.4. Data Extraction and Synthesis

To eliminate potential bias during the selection process, four authors (M. Sh., M. D., V. U., and M. M.) independently conducted data extraction, initially screening titles and abstracts to exclude irrelevant studies. Full-text reviews were then performed on the remaining studies to determine their final inclusion. Any discrepancies in selection or data interpretation were resolved through discussion with the corresponding author (F. M. and K.Y.R.). M. Sh., and M. M. collected and verified data using a standardized form. If any uncertainties arose, the corresponding author provided the final decision.

Any missing summary statistics (e.g., sensitivity, specificity, or the limit of detection) were documented as “not available” to maintain transparency. No statistical imputation or data conversion was performed, as only clearly reported numerical values from each study were used. The extracted data were organized and tabulated in structured summary tables to display individual study characteristics, including study authors, publication year, research design, biosensor type, target pathogen (virus or bacteria), detection methodology, and performance metrics such as sensitivity, specificity, and the limit of detection. For synthesis, studies were grouped and compared based on shared characteristics, such as the biosensor type, target pathogen category, detection principles, and diagnostic performance metrics.

To explore potential sources of heterogeneity among the included studies, subgroup comparisons were conducted narratively across variables such as biosensor type, pathogen type, and detection methodology. However, due to the qualitative nature of the synthesis, no statistical tests for heterogeneity were applicable. Sensitivity analyses were also not applicable in this systematic review as no pooled estimates or meta-analytic calculations were performed.

### 2.5. Certainty Assessment

The certainty of the body of evidence for each outcome was assessed based on several factors, including the consistency of findings across studies, methodological quality, clarity of outcome reporting, and relevance to the review objectives. Greater confidence was attributed to findings supported by multiple high-quality studies using comparable biosensor technologies and reporting consistent diagnostic performance metrics. Studies with unclear methodologies, small sample sizes, or missing performance data were considered to provide lower certainty evidence.

### 2.6. Ethical Statement

As this is a systematic review, registration with a Research Ethics Committee (REC) was not required. Additionally, all supporting data referenced in this review are publicly accessible within the cited studies.

## 3. Results

### 3.1. Structure–Property–Function Relationships

Noble MNCs exhibit molecular-like properties together with quantum confinement effects leading to a strong luminescence without any surface plasmon absorption peak compared to the larger MNPs. MNCs usually consist of a metal core (i.e., Cu, Ag, Au, Pt) and a ligand shell [50]. Notably, their fluorescence is sensitive to the microenvironment which is affected by the electron-transferring changes between the protected ligand and the metal core. In this way, the synthesis method is an important factor. Fluorescent MNCs are usually synthesized based on top-down and bottom-up approaches. In the top-down method, after metal nanoparticle formation, they are etched at a high temperature. Chemical techniques can also be effective through direct core reduction and interface etching using high concentrations of thiol ligands (e.g., 11-Mercaptoundecanoic acid and GSH) at a high pH (>12.0) or under blue LED irradiation [51].

In the bottom-up method, metal ions (e.g., Au^3+^, Cu^2+^, Ag^+^, Pt^4+^) are reduced to zero metal atoms [52] using various reduction methods such as chemical reduction, photoreduction, microwave reduction, electrochemical reduction, and sonochemical reduction. This method causes atom-by-atom construction, allowing homogeneous and ultra-small NCs with fewer defects. In this way, a capping agent can act as both a reducing and a stabilizing agent and therefore there is no need to use a reducing agent. Otherwise, a reducing agent should be used such as sodium hypophosphite or sodium borohydride. Moreover, in the photoreduction method, visible and UV lights are used for MNC synthesis without any use of reducing agents. In sonochemical reduction, MNCs can be produced under ultrasonic irradiation. Microwave-assisted reduction is a fast and simple approach with high reproducibility, and the electrochemical reduction method forms MNCs with uniform sizes. Also, direct laser writing (DLW) has recently been introduced as a novel bottom-up method for patterning and synthesizing MNCs to create solid-state devices for different applications [50,53].

Key parameters to control the structure–property–function, size, and oxidation state which affect the optical properties of MNCs include the following: the types of capping agents and reducing agents, the metal ion species and their concentrations, the pH of the solution, the reaction time, and the temperature. For example, red-emitting Pt16NCs and blue-emitting Pt6NCs were simultaneously synthesized at pH 12 and 37 °C by hemoglobin (Hb) with a [Hb]/[Pt] ratio of 0.88 [54]. Moreover, the blue-emitting Ag_5–7_NCs at pH 9 and the red-emitting Ag_28_NCs at pH 12 were prepared using the same peptide. Also, Ag_15_NCs and Ag_5_NCs were stabilized on cytosine-rich ATP1 DNA at room temperature [55]. Moreover, the effect of temperature on reaction rates and MNC size is demonstrated in the synthesis of BSA-AuNCs (reduced from 12 h to 20 min) and Hb-AgNCs (from 21 to 10 days) when temperature increases from 37 °C to 70 °C [56].

Other important parameters affecting the lifetime, emission color, size, and quantum yield of MNCs include the following: (i) For proteins, protein structural flexibility, amino acid composition, and ratios of tyrosine/tryptophan and amine/thiol are critical for synthesizing stable MNCs. Tyrosine residues with phenolic groups reduce Au^1+^ to AuNCs in alkaline media. (ii) For peptide templates, amino acid composition and length, especially the Cys-Cys-Tyr (CCY) fragment, play a key role. In this regard, the designed peptide typically consists of a functional sequence for targeting, a directing fragment for MNC formation, and a linker part connecting the recognition sequence to the directing part. (iii) For DNA capping agents, the length and base composition of the DNA, the MNC-stabilizing sequence, cytosine’s presence at the 3′ end of MNC-stabilizing sequences, thymine-rich targeting fragments, the stem length, the distance between the stem and loop structures, and the cytosine number in the loop significantly influence the optical properties of DNA-MNCs [57,58].

### 3.2. Types of MNC-Based Biosensors

There are several types of MNC-based biosensors that utilize different detection mechanisms, based on NCs’ properties such as optical, electrochemical, and catalytic characteristics [59,60].

Optical biosensors operate by detecting alterations in the optical signal caused by biological agents, such as bacteria or viruses. These sensors typically employ a range of spectroscopic techniques, including absorption, fluorescence, Raman scattering, and refraction, to measure changes in light properties. Common examples include fluorescence sensors, colorimetric sensors, optical fiber sensors, and surface plasmon resonance (SPR) sensors. They are characterized by high sensitivity, selectivity, and versatility in detecting biological interactions [61,62,63].

Fluorescence-based optical biosensors are widely employed for the detection of different analytes due to their high accuracy, sensitivity, and affordable, straightforward applications [64]. Several key features of these biosensors, including emission intensity, quantum yield, energy transfer, and fluorescence lifetime, can be effectively employed for analyte detection [65]. Unlike larger metal NPs that exhibit SPR effects, MNCs exhibit stable fluorescence, making them a highly promising platform for rapid and label-free biosensing [34,59]. Generally, in MNC-based fluorescence biosensors, detection relies on the interaction between the target analyte and MNCs, resulting in measurable fluorescence changes. These changes are based on fluorescence enhancement (signal-on) [66], fluorescence quenching (signal-off) [67], shifts in the emission wavelength [68], fluorescence resonance energy transfer (FRET) [69], or fluorescence switching (off–on, on–off) [70].

Colorimetric biosensors are another type of optical biosensor that generate a visible color change, allowing detection without advanced instruments. They are simple, cost-effective, and real-time methods that enable the easy identification of target molecules, even with the naked eye [71,72,73]. The three primary types of colorimetric biosensors, categorized based on factors influencing their color response [74], include non-nanomaterial-based biosensors whose color change depends on external factors such as temperature [75], or pH [76]; enzyme-catalyzed biosensors [77] that rely on enzymatic reactions but are susceptible to environmental conditions (which can be improved by enzyme-like nanomaterials); and metal-based nanomaterial biosensors, where color changes occur through aggregation or dispersion [50]. MNC-based nanoplatforms have been extensively explored for colorimetric biosensors because MNCs exhibit strong catalytic activity, high water solubility, facile synthesis, biocompatibility, and favorable surface chemistry for efficient conjugation [78,79]. MNC-based colorimetric biosensors primarily function through their intrinsic enzyme-like properties, with peroxidase-like activity as the predominant mechanism. Most of them oxidize substrates such as 3,3′,5,5′-tetramethylbenzidine (TMB) in the presence of hydrogen peroxide (H_2_O_2_). A catalytic reaction generates a colorimetric signal through a color change or by increasing, decreasing, or recovering peroxidase-like activity, which can be characterized using an ultraviolet–visible spectrophotometer or with the naked eye [26,50].

Electrochemical biosensors integrate the analyte-recognition component with an electrochemical transducer. The interaction between the analyte and the transducer yields a measurable electrochemical signal, which can manifest as changes in current, voltage, impedance, or resistance. These biosensors offer fast response times, high sensitivity, cost-effectiveness, and portability, eliminating the need for centralized laboratory facilities [80,81,82]. MNC-based electrochemical biosensors utilize the distinctive electrical and catalytic properties, atomic-modulated structures, high conductivity, and large specific surface areas of MNCs. These properties enable MNCs to facilitate electron transfer or act as redox mediators, advancing the field of electrochemical biosensing [83,84].

Therefore, biosensors designed for pathogen detection can be categorized based on the type of transducer used, such as optical, electrochemical, and others. Alternatively, they can also be classified according to the type of bioreceptor employed, including immunosensors (relying on antibody–antigen interactions), aptasensors (employing DNA or RNA aptamers), genosensors (using DNA probes), and others [81,85,86]. The integration of nanotechnology into biosensor development has led to significant improvements in their efficiency and sensitivity [24].

#### Metal Nanoclusters in Point-of-Care Sensing

MNCs are promising materials in point-of-care (POC) diagnostics for pathogen sensing due to their unique optical, electrochemical, and catalytic properties leading them to act as highly sensitive and accurate probes in a short time. Among MNCs, AuNCs, AgNCs, CuNCs, and PtNCs are interesting in POC applications due to their desirable quantum yields and reversible oxidative behaviors. For example, Ji et al. designed four protein–AuNCs (i.e., BSA-AuNCs, HSA-AuNCs, lactoferrin-AuNCs, and lysozyme-AuNCs) as a fluorescence sensor array for the rapid detection of six kinds of bacteria including *S. aureus*, MRSA, *E. coli*, KREC, *B. subtilis*, and *A. faecalis*. The strategy exhibited the advantages of convenient and easy synthesis with excellent classification accuracy. Various bacteria surfaces showed different interactions with the sensor array with respect to the changes existing in their physicochemical nature leading to the generation of fingerprint-like patterns [87]. AuNCs with excellent properties such as enzyme-mimicking activity and high functionality are also promising candidates for designing electrochemical point-of-care architectures. In addition, AuNCs are much more biocompatible compared to conventional electrochemical redox agents (such as K_4_[Fe (CN)_6_]). For example, the determination of H_2_O_2_ and glucose has been reported with a higher performance using AuNCs. However, one limitation of AuNCs compared to conventional redox reagents is their low electrochemical signal. This explains the limited studies on electrochemical POC applications using AuNCs in the literature compared to AuNC-based optical sensors. Therefore, novel AuNCs with high electrochemical signals need to be designed in the future. Most optical POC biosensors with AuNCs focus on chemiluminescence (CL) and electrochemiluminescence (ECL). Considering the cost-effectiveness and simplicity of CL systems, along with AuNCs’ excellent properties, these systems provide POC sensing opportunities [26]. The CL-enhancing behavior of AuNCs can be achieved by their catalytic activity and by energy transfer from a luminophore to AuNCs. For example, Li et al. reported a CL resonance energy transfer from bis(2,4,5-trichloro-6-carbopentoxyphenyl) oxalate (CPPO)/H_2_O_2_ as a donor to aggregated AuNCs as acceptors for CN^−^ detection. In this work, AuNCs also catalytically enhanced the reaction rate between CPPO and H_2_O_2_ [88]. In fact, CL is an interesting POC strategy for the on-site, rapid, real-time, cost-effective, and sensitive detection of different markers for chemical and biomedical analysis. CL systems do not require the use of light or potential. In addition, various nanostructures can be used in CL platforms as emission enhancers, emitters, and quenchers [89]. The ECL-based POC method is powerful with respect to its high selectivity, excellent sensitivity, and broad dynamic linear range. MNCs could increase the ECL signals in two ways: (i) MNCs act as electrosensitizers to enhance the electron transfer rate thorough producing electrogenerated holes on the electrode surface; (ii) the generation of active radicals allows the production of more luminophores and thus the co-reactant acceleration mechanism. In ECL applications of MNCs, one fundamental problem is slow electron transfer during redox reactions due to the low-conductivity stabilizers, resulting in a low ECL quantum yield. Therefore, designing electrochemiluminophores with high electron-transfer ability is needed [90]. For instance, Zhu et al. reported an ECL system for GSH determination based on Cu_2_O and AuNCs in which Cu_2_O acts as an electrosensitizer to produce electrons in AuNCs. These electrons migrate from the HOMO of AuNCs to the valence of Cu_2_O and then to the electrode surface. This finally causes an increase in the ECL intensity of AuNCs [91].

AgNCs with unique physicochemical properties such as well-defined small size and intense emission have attracted attention in POC applications. Nevertheless, zero-valent Ag indicates a high reactivity challenge so that it could be easily oxidized during its synthesis. Since AgNCs are considered soft acids, soft ligands such as amine molecules and thiols can be used to stabilize their structures. Notably, among stabilizing agents, DNA-stabilized/AgNCs have presented superior luminescence characteristics upon hybridization with different targets (e.g., DNA and RNA). In one strategy, G-rich lighting-up sequences could be used in a recognition target sequence so that the dark or weak-emissive DNA-AgNCs could be highly lit up by approaching guanine [92]. However, G-rich reporters require precise design. In other strategies, luminescence enhancement or quenching happens under the self-assembly of DNA-AgNCs on GO nanosheets so that in the presence of the target, the optical behaviors are reversed [93].

AgNCs have also been proposed for alloying and doping with other noble MNCs (e.g., Au, Pt, and Pd) to improve their performance, signaling, strength, and stability with comparable diameters to Ag in the POC surfaces [94]. Doped atoms are usually inside the core of AgNCs [95]. Linden et al. showed that the quantum yield increased about 3–4 times by doping Au atoms into Ag_29_NCs, but the emission lifetime decreased from 4 to 2.6 μs. Moreover, with a low concentration of Au, highly stable Au/AgNCs were synthesized [95]. Results have demonstrated that noble metal doping can also amplify the ECL similarly to that of PL [96]. These advantages have caused doped Au/AgNCs to be considered for diagnostic purposes in the past several years [97].

The alloy MNC probes boost the PL properties and can be generated by easily mixing the Au and Ag precursors in the presence of thiol ligands such as GSH S-transferase (GST) as a reducing and stabilizing agent in alkaline media. These alloyed Au/AgNC probes could produce two emissions which is desirable in ratiometric detection. After Au/AgNCs, CuNCs are the most attractive MNCs, even better than AgNCs. This could be attributed to their large-scale applications [26]. However, control of CuNCs’ size is difficult since they are very sensitive to oxygen and air [98]. Another challenge in relation to CuNCs is the low quantum yields in the IR region. Therefore, many attempts have been made to design novel synthesis methods to prepare improved stable CuNCs with high emission quality. One strategy to solve these problems is using MOFs with three-dimensional and electron-rich structures. In this regard, CuNCs can be doped into highly rigid and stable structure of MOFs. Therefore, given their reactivity with air, the amount of oxygen decreases. For example, Jana et al. synthesized three thiolated carborane-stabilized CuNCs with various ligands including yellow-emitting Cu4@ICBT and green-emitting Cu4@mCBT and Cu4@oCBT that exhibit quantum yields of 18%, 59%, and 81% [99]. The modification of CuNCs with β-CD is another desirable approach to prepare stable CuNCs [100]. Among different MNCs, PtNCs have received less attention. This may be due to the fact that the specific advantages of PtNCs in POC applications are yet unknown.

### 3.3. MNC-Based Biosensors for the Detection of Bacteria

#### 3.3.1. Colorimetric-Based Optical Nanobiosensors

##### AuNCs

**Figure 2:**
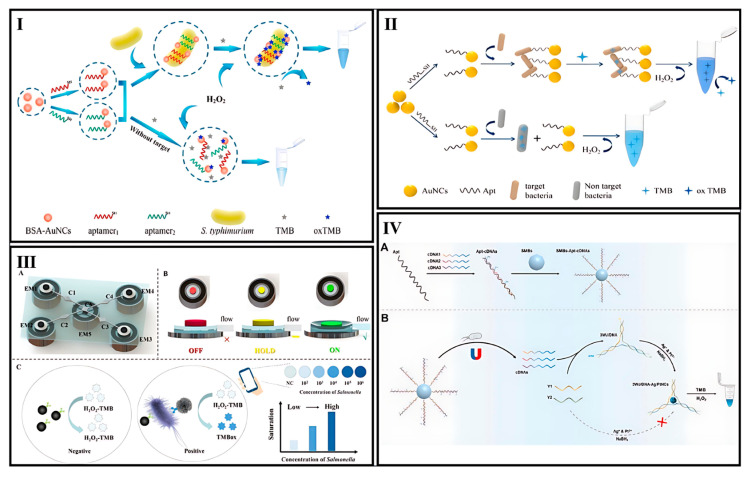
(**I**) Diagram showing the working principle of a colorimetric aptasensor for detecting *S. typhimurium* [103]. (**II**) Illustration of the detection mechanism using an aptamer-papain-AuNCs sensor [105]. (**III**) Principle of a *Salmonella* colorimetric biosensor: (**A**) biosensor structure composed of C1-C5 chambers, (**B**) electromagnetic states for fluid control, (**C**) sensing mechanism [108]. (**IV**) Illustration of a colorimetric biosensor for *S. typhimurium* detection in milk, using aptamer-induced multi-DNA release and peroxidase-mimicking DNA-Ag/PtNCs: (**A**) Illustration of the design of biosensor, (**B**) Illustration of the detection mechanism [111].

AuNCs have been widely explored for bacterial detection due to their excellent peroxidase-like activity [101,102]. The peroxidase-like property of AuNCs was used as a colorimetric strategy for biosensor designs. Several designs based on thiolated aptamer-modified AuNCs have been reported for bacteria detection. In a study conducted by Chen et al., thiolated dual aptamers modified with bovine serum albumin stabilized-gold nanoclusters (aptamers@BSA-AuNCs) were developed for *Salmonella typhimurium* (*S. typhimurium*) detection. The peroxidase-like activity of aptamers@BSA-AuNCs in the presence of micro-sized bacteria increases and thus converts colorless TMB to blue oxidized TMB (ox-TMB). As a result, the promoted formation of blue products in the catalytic system was used for *S. typhimurium* colorimetric detection. Its practical applicability was confirmed through the identification of *S. typhimurium* in eggshell and egg white samples, achieving recovery rates ranging from 92.4% to 110% [103] (Figure 2I). In a study conducted by Xie et al., an enzyme mimicking a AuNC–chitosan composite membrane accompanied with aptamers and AuNPs was developed for the selective detection of *Staphylococcus aureus* (*S. aureus*). AuNCs behave as peroxidase enzymes and can catalyze the reaction of H_2_O_2_ and TMB, displaying the blue color of the ox-TMB. AuNPs were further used for naked-eye readouts based on color shifts. In this manner, H_2_O_2_ reacts with HAuCl_4_ to form AuNPs, with sufficient H_2_O_2_ producing the dispersed wine-red AuNPs and insufficient H_2_O_2_ yielding blue/violet aggregated AuNPs. The AuNC–chitosan membrane catalyzes H_2_O_2_ decomposition, reducing its availability for AuNP formation. In the presence of the target, specific reactions block the membrane’s catalytic sites, altering H_2_O_2_ levels and resulting in color changes (wine-red to blue/violet) for visual detection. This design was reported for rapid onsite pathogenic bacteria screening and enterotoxin detection for food safety and environmental monitoring [104]. *Escherichia coli* (*E. coli*) O157:H7 was another target detected based on AuNCs’ peroxidase activity. Song et al. developed aptamer-modified papain-AuNCs with promoted peroxidase activity for *E. coli* O157:H7 detection based on TMB. *E. coli* O157:H7 was quantitatively detected in pure culture at 39 CFU/mL. The detection limits were reported as 5.6 × 10^2^ CFU/mL in ultra-high-temperature sterilized milk, 5 × 10^2^ CFU/mL in pasteurized milk, and 4.9 × 10^2^ CFU/mL in raw milk samples [105] (Figure 2II). AuNCs’ peroxidase activity was recently used as an in vivo bacterial detection method. In a study conducted by Chen et al., renal-clearable AuNCs with enhanced catalytic activity (LOD of 0.028 pmol) were developed for in vivo *S. aureus* detection as a major contributor to hospital-acquired infections related to implants. AuNCs were encapsulated in bacterial toxin-responsive liposomes. The biosensor operated by releasing AuNCs from liposomes when encountering *S. aureus* toxins which produced a detectable color change after undergoing kidney-mimetic filtration. A hyaluronic acid (HA) hydrogel implant infection model was used to further validate the biosensor both in vitro and in vivo. In mice with infected HA hydrogel implants, their urine changes to blue upon substrate exposure, suggesting this method could enable non-invasive implant-related infection monitoring [106].

##### AgNCs

Aptamer-modified magnetic beads AuNPs were employed for *Listeria monocytogenes* (*L. monocytogenes*) colorimetric detection in a project conducted by Liu et al. They developed a colorimetric immunoassay based on the *o*-phenylenediamine (OPD)-mediated deaggregation of AuNPs with IgY-conjugated AgNCs. At first, AuNPs were aggregated by OPD. In the presence of the target, sandwich complexes form by using aptamer-modified magnetic beads and the IgY-coated AgNCs. Then AgNCs convert OPD to oxidized OPD, triggering the deaggregation of AuNPs and causing a color change from blue to red. This color shift correspond to the bacterial concentration, enabling simple and visual detection after magnetic separation with an LOD of 10 CFU/mL and recovery from 97.4 to 101.3% in spiked food samples [107].

##### Manganese Dioxide NCs

Manganese dioxide NCs were also used for pathogenic detection. Lu et al. developed a colorimetric biosensor for the automated detection of *Salmonella* in a sealed microfluidic chip, designed for point-of-care testing to prevent food poisoning. The chip design included a central mixing chamber for immunomagnetic nanoparticles (IMNPs), bacterial samples, and immune manganese dioxide nanoclusters (IMONCs), along with dedicated chambers for reagents and fluid control. Four electromagnets manipulated iron cylinders to precisely control fluid flow, enabling automated mixing, magnetic separation, washing, and catalytic reactions. IMNP-bacteria-IMONC conjugates were formed, separated, and resuspended in an H_2_O_2_-TMB substrate, where IMONCs catalyzed a color change from colorless to blue. The resulting color was analyzed via a smartphone app to determine bacterial concentration based on enhanced peroxidase-like activity with a low detection limit of 101 CFU/mL in 30 min [108] (Figure 2III).

##### Bimetallic NCs

Bimetallic NCs were also used as a colorimetric bacterial detection probe based on their peroxidase activity on the TMB reagent. Au/Pt NCs were synthesized on the cytosine nucleotide end of a 30-mer DNA probe based on the hipo gene of *Campylobacter jejuni* (*C. jejuni*) by Dehsghani et al. The linear decrease in peroxidase activity of Au/Pt NCs upon the addition of bacterial target DNA was used as the detection signal. An LOD of 20 pM was reported for this bacterial DNA sensor. The approach effectively detected bacterial target DNA in milk samples [109]. In another study, researchers reported DNA-Au/Pt NCs as natural enzymes for *S. aureus* detection in food samples. This paper-based design demonstrated a broad linear dynamic range, effectively detecting *S. aureus* concentrations spanning from 10^8^ to 10^2^ CFU/mL, with an LOD as low as 80 CFU/mL. In this work, TMB is converted to ox-TMB by the bimetallic NC probe in the absence of the target. After the addition of the bacterial target, ox-TMB, and thus the blue color, is not produced as a result of the decreased peroxidase-like activity of the designed probe [110]. Ag/Pt NCs were also employed for colorimetric bacterial detection. In a study conducted by Pang et al., the Ag/Pt NCs were synthesized on a three-way junction DNA template (3WJ/DNA-Ag/Pt NCs) for *S. typhimurium* detection with the help of aptamer-functionalized magnetic beads (SMBs-Apt). In this design, the aptamer bound to three complementary DNAs (cDNAs) to form Apt–cDNA complexes, which were linked to magnetic beads via streptavidin/biotin interaction. In the presence of *S. typhimurium*, the aptamer bound to it, releasing the cDNAs into the supernatant after magnetic separation. These cDNAs, along with added DNAs (Y1 and Y2), formed a three-way junction (3WJ) DNA structure. This 3WJ/DNA template enabled the synthesis of DNA-Ag/Pt NCs with peroxidase-like activity, catalyzing a TMB–H_2_O_2_ reaction to produce a blue color. Without the target pathogen, the 3WJ/DNA structure could not form, preventing the synthesis of Ag/Pt NCs and the color change [111] (Figure 2IV).

##### Fe-NCs

Zhang et al. developed a capillary biosensor for the rapid and sensitive detection of *S. typhimurium*, aiming to enable the early screening of foodborne pathogens. The biosensor used a multi-column capillary preloaded with magnetic nanoparticles (MNPs), phosphate wash buffer containing Tween 20 (PBST), Fe-NCs for signal amplification, and HCl, separated by air gaps. An iron spiral mixer and multi-ring magnets facilitated the efficient mixing and transfer of MNPs and their conjugates. Target bacteria were captured by MNPs, conjugated with Fe-NCs, and washed before transferring to HCl, where released iron ions reacted to form Prussian Blue. The color change was analyzed using a smartphone app based on Hue–Saturation–Lightness color space [48]. This biosensor with the potential for foodborne pathogen detection exhibited a recovery of ~105.0% in spiked chicken samples with an LOD of 14 CFU/mL.

#### 3.3.2. Fluorescence-Based Optical Nanobiosensors

##### AuNCs

**Figure 3:**
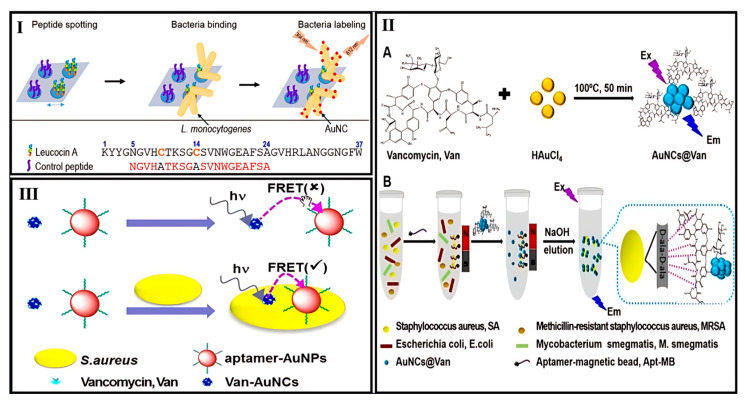
(**I**) Schematic representation of *Listeria monocytogenes* detection using LeuA and AuNCs [114]. (**II**) Schematic representation of (**A**) single-step synthesis of vancomycin-conjugated gold nanoclusters (AuNCs@Van) and (**B**) dual-recognition detection strategy for Staphylococcus aureus (SA) in mixed samples using Apt-MB and AuNCs@Van [116]. (**III**) Visual representation of the FRET-based detection platform for *S. aureus* employing dual-recognition elements: vancomycin and aptamer [118].

Traditionally, microbiological assays are used to identify the bacterial strains responsible for infection. However, these assays can be time-consuming, often taking several days to yield results. Therefore, reducing the time required for these assays is beneficial for enhancing medical diagnostics and treatment outcomes. In this way, fluorescent nanobiosensors based on AuNCs are suitable because of their unique luminescence properties, ease of functionalization and synthesis, high photostability, and small sizes. In a study conducted by Chan P-H et al., glycan-bound nanoprobes were recognized as effective biosensing tools for detecting bacteria with glycan-binding sites. As a model system, mannose-capped gold nanoclusters (AuNCs@Mann) were employed for *E. coli* J96 detection in urine. The selectivity of this study is based on the ability of AuNCs@Mann to specifically bind to bacteria containing mannose-binding sites, allowing the direct visualization of bacterial aggregation. Additionally, the fluorescence intensity of the supernatant decreased with increasing *E. coli* J96 concentration, as more AuNCs@Mann bound to the bacterial cells and were sedimented during centrifugation. The results showed that the AuNCs@Mann are aggregated in the presence of *E. coli* J96, resulting in an aggregation-caused quenching (ACQ) mechanism with 2 × 10^6^ cells mL^−1^ [112]. Khlebtsov et al. synthesized highly fluorescent BSA-AuNCs functionalized with anti-staphylococcal human immunoglobulin (anti-SAIgG) and the photodynamic agent Photosens^TM^ (PS), forming Au–BSA–anti-SAIgG–PS complexes. These probes, due to their high biospecificity and intense fluorescence, enable the detection of pathogenic bacteria in complex mixtures via fluorescence microscopy or direct visual inspection under UV light. Additionally, photodynamic treatment of *S. aureus* with these complexes under 660 nm light irradiation resulted in significant bacterial inactivation [113]. *L. monocytogenes* is a Gram-positive bacterium commonly associated with foodborne infections, including listeriosis and meningitis. Due to its high mortality rate (20–40%), this pathogen represents a major public health concern. In one study, a peptide-based biosensor was introduced for the detection of *L. monocytogenes* in contaminated food samples. The detection platform utilizes Leucocin A (LeuA), an antimicrobial peptide with high specificity for *Listeria* species, enabling targeted binding to the bacteria. To facilitate bacterial capture, LeuA is immobilized onto a glass surface, forming a self-assembled monolayer (SAM). When a contaminated sample is introduced, *L. monocytogenes* selectively binds to the peptide-coated surface. Additionally, 3-mercaptopropionic acid (MPA)-stabilized AuNCs are synthesized in situ on the glass surface, directly on the bacteria-bound region. The resulting MPA-AuNCs exhibit strong fluorescence at 612 nm, enabling detection of the pathogen [114] (Figure 3I). A selective fluorescence detection method for *E. coli* O157:H7 was also developed by integrating chitosan (CS) nanocapsules embedded with glutathione-stabilized AuNCs with immunological recognition and magnetic separation. The AuNCs@CS nanocapsules enable the encapsulation of a large number of fluorescent AuNCs within each nanocapsule, creating a signal amplification system compared to the AuNCs. In addition, for specific detection, IMNPs and immunofluorescent nanocapsules were developed by conjugating the anti-*E. coli* O157:H7 antibody onto the surfaces of MNPs and AuNCs@CS nanocapsules, respectively. Following magnetic separation, the bacteria were quantitatively determined by measuring the fluorescence (at 572 nm) of the AuNCs@CS nanocapsules attached to the bacterial cells, which increased with the increase in target bacteria concentration. In this study, a linear range was found in the range of 3–700 CFU/mL, with an LOD of 1 CFU/mL [115]. In another study, Dan Cheng et al. developed a strategy utilizing dual recognition elements (antibiotic and aptamer), combined with magnetic enrichment and a fluorescence assay, to accurately quantify *S. aureus* in complex samples, even when other bacteria were present in higher concentrations. The selectivity of this method was achieved by integrating aptamer–magnetic beads (Apt-MBs) and Vancomycin–gold nanoclusters (Van-AuNCs) for the specific detection of *S. aureus*. The blue emission of Van-AuNCs increased proportionally with the concentration of the target bacteria. This developed antibiotic- and aptamer-based dual recognition strategy was widely available and much less expensive compared to commonly used antibodies [116] (Figure 3II). Another study reported that human serum albumin–gold nanoclusters (HSA-AuNCs) exhibit high selectivity for *S. aureus* and methicillin-resistant *S. aureus* (MRSA). Experimental findings revealed that target bacteria detected by HSA-AuNCs, which emit reddish fluorescence, are easily visible after centrifugation. The peptide motifs of HSA-AuNCs with a binding affinity to *S. aureus* and MRSA were also characterized and identified by mass spectrometry and used to synthesize AuNCs (Pep-AuNCs) under microwave heating. The Pep-AuNCs, similar to the HAS-AuNCs, were applied as a sensing probe for *S. aureus* and MRSA [117]. Mengqun Yu et al. developed a universal one-step method for the selective and sensitive detection of *S. aureus* using a dual molecular affinity-based FRET strategy. This method relies on the concurrent targeting of the bacterial cell wall by both antibiotic and aptamer molecules. Within 30 min, using Van-functionalized AuNCs as the energy donor and aptamer-modified AuNPs as the energy acceptor, the FRET signal exhibited a linear response to *S. aureus* concentrations ranging from 20 to 10^8^ CFU/mL, with a detection limit of 10 CFU/mL. The approach demonstrated high specificity, as non-target bacteria yielded negative results. The unique feature of this article regarding fluorescence is the use of a dual recognition FRET platform, combining blue-emitting Van-AuNCs as the energy donor and aptamer-modified AuNPs as the energy acceptor [118] (Figure 3III). S.S. Evstigneeva et al. published a study in 2023, Bacterial biofilms frequently colonize chronic wounds and the surfaces of medical devices, highlighting the need for reliable imaging and detection methods. While fluorescence-based bacterial identification is both sensitive and non-destructive, its application to biofilm detection remains limited due to the lack of biofilm-specific fluorescent dyes. This study presents the first evidence that glutathione–gold nanoclusters (GSH-AuNCs), in the absence of targeting ligands, selectively bind to the extracellular matrix components of bacterial biofilms, both Gram-negative and Gram-positive, resulting in fluorescent biofilm labeling. In contrast, other AuNCs, including BSA-AuNCs and 11-mercaptoundecanoic acid-stabilized gold nanoclusters (MUA-AuNCs), do not exhibit extracellular matrix staining. Molecular docking studies revealed that GSH-AuNCs have an affinity for several extracellular matrix targets, including amyloid-anchoring proteins, matrix proteins, and polysaccharides. Experimental results provided evidence of GSH-AuNC interaction with lipopolysaccharide (LPS) isolated from the extracellular matrix of *Azospirillum baldaniorum* biofilms. Based on these properties, a novel fluorescent method was developed for quantitative biofilm biomass measurement, achieving an LOD of 1.7 × 10^5^ CFU/mL—a 10-fold improvement over the standard crystal violet assay. A strong linear correlation was observed between biofilm fluorescence intensity and bacterial count within the range of 2.6 × 10^5^ to 6.7 × 10^7^ CFU/mL. This article uniquely demonstrates that GSH-AuNCs can selectively bind to bacterial biofilm components without targeting ligands, enabling highly specific fluorescent staining. It also reveals an aggregation-induced fluorescence enhancement (AIE) mechanism upon interaction with biofilm LPS, making GSH-AuNCs a more sensitive biofilm detection tool than conventional methods. This methodology enabled a quantitative assessment of biofilm formation on urinary catheter surfaces, demonstrating its potential for diagnosing medical device-associated infections [119].

##### AgNCs

*Salmonella* is one of the most significant pathogens and a leading cause of foodborne diseases worldwide. Peng Zhang et al. developed a biosensor for directly detecting live *S. typhimurium*. This research introduces a system that utilizes a designed triple-trigger sequence-regenerated strand displacement amplification (SDA) mechanism, combined with a self-protective hairpin template for scaffolded AgNC formation. In the presence of viable *S. typhimurium*, single-stranded trigger sequences are released from the aptamer–trigger sequence complex, which initiate a branch migration process that unfolds Hairpin Template I, which contains complementary scaffolds for AgNC synthesis. This event triggers the first round of SDA, generating a significant number of AgNC scaffolds and reporter strands. These newly synthesized reporter strands then initiate another branch migration, opening Hairpin Template II, which also harbors complementary AgNC scaffolds. Subsequently, the second SDA cycle produces additional AgNC scaffolds and trigger sequences, leading to a third strand displacement and amplification event. This cyclic amplification mechanism enables the continuous regeneration of trigger sequences and the sequential production of AgNC scaffolds, resulting in the formation of highly fluorescent AgNCs. The enhanced fluorescence signal facilitates the ultrasensitive detection of viable *S. typhimurium*, achieving a detection limit as low as 50 CFU/mL, with a linear detection range spanning from 10^2^ to 10^7^ CFU/mL.

This method achieves fluorescent detection of *S. typhimurium* without requiring labeling, probe modification, or DNA extraction steps. The method significantly enhances fluorescence intensity through cyclic amplification, achieving ultrasensitive detection down to 50 CFU/mL while distinguishing live bacteria through heat. This work is an instance of a fluorescent biosensor capable of directly distinguishing live *S. typhimurium* from heat-denatured counterparts, while also introducing a novel strategy for DNA-scaffolded AgNC generation. The authors suggest that this biosensor could be adapted into a versatile platform for directly detecting various viable pathogenic bacteria by modifying the corresponding aptamers as recognition probes [120]. In another study, researchers proposed a fluorometric approach for detecting *S. typhimurium*. This approach integrates target-modulated photoinduced electron transfer (PET) between G-quadruplex DNAzyme and DNA-AgNCs, combined with a circular exponential amplification mechanism based on hairpin probes. This mechanism efficiently quenches fluorescence, enabling the ultrasensitive detection of *S. typhimurium*. Additionally, the circular exponential amplification strategy enhances signal sensitivity while maintaining high specificity. The reaction system consists of three hairpin probes (H1, H2, and H3), each serving a distinct function. Probe H1 contains an aptamer specific to *S. typhimurium* along with a recognition sequence for nicking endonuclease. It plays a crucial role in recognizing *S. typhimurium* and facilitates both polymerase-driven target recycles amplification and secondary target recycle amplification. Probe H2 includes an aptamer for hemin, enabling the formation of a G-quadruplex DNAzyme in the presence of hemin and potassium ions. This DNAzyme functions as an electron acceptor, effectively quenching the fluorescence of labeled DNA. The optimal excitation/emission wavelengths for fluorescence measurement are 567/650 nm. Probe H3 contains the template sequence necessary for AgNC synthesis and the H2-annealing sequence. Both H2 and H3 participate in a strand displacement reaction, facilitating PET between G-quadruplex DNAzyme and DNA/AgNCs [121]. Jiamei Zhang et al. conducted a study aimed at developing a novel method for the quantitative detection of *E. coli* in food using DNA-AgNCs. Initially, an immunomagnetic nanoprobe was synthesized by conjugating MNPs with an antibody specifically targeting *E. coli* O157:H7. Subsequently, aptamer-modified AgNCs were introduced to form silver nanoprobes, resulting in a sandwich composite structure. Upon binding of the AgNC probes to magnetically captured *E. coli*, unbound silver nanoprobes remained in the supernatant. The bacterial concentration in the sample was then indirectly determined by measuring the fluorescence intensity of the supernatant. A distinctive aspect of this study is the application of BSA-AgNCs conjugated with aptamers (BSA-AgNCs-Apt) to achieve the specific detection of *E. coli* O157:H7. The method exhibited a linear detection range of 10 to 10^6^ CFU/mL, with a detection limit of 0.2549 CFU/mL in buffer and 0.6031 CFU/mL in a milk-based simulation [122]. In another study in 2022, researchers introduced a fluorescence-based aptasensor for detecting *S. typhimurium* utilizing DNA-AgNCs. By integrating magnetic beads, aptamers, and AuNPs, a sandwich-structured fluorescence quenching system was developed. In the presence of target bacteria, the system was disrupted, resulting in a significant increase in fluorescence intensity. At first, the streptavidin-modified magnetic beads–cDNA1 (SMBs-cDNA1) complex, the AuNPs-cDNA2, and the aptamer were mixed, resulting in fluorescence quenching. After the addition of *Salmonella*, the aptamer specifically connected to the target, allowing the discharge of AuNPs-cDNA2 and fluorescence enhancement. Therefore, in the absence of *S. typhimurium*, the SMBs-Apt-AuNPs quenched the fluorescence of DNA-AgNCs. The proposed system exhibits specificity through fluorescence quenching and restoration mechanisms (turn-on), enabling the rapid (within 2 h) detection of *S. typhimurium* [123].

In another study by Xiaodong, a simple and effective approach was developed using guanine-rich (G-rich) DNA sequences to construct a comprehensive library of logic gates on a DNA-AgNCs platform. The library included YES, AND, OR, INHIBIT, and XOR, which were further combined into complex logic circuits to implement various advanced arithmetic and non-arithmetic functions such as half-adder, half-subtractor, multiplexer, and demultiplexer. Upon UV irradiation, all logic functions were immediately visible, demonstrating exceptional reproducibility. The logic operations relied entirely on DNA hybridization under enzyme-free and label-free conditions, minimizing waste generation and lowering costs. Notably, the DNA-AgNC-based multiplexer was employed as an intelligent biosensor for the highly sensitive detection of pathogenic genes, specifically *E. coli* and *S. aureus*. The results showed a high fluorescence signal only when a specific bacterial gene was present. The multiplexer could transfer the *S. aureus* gene state into the output channel; if so, the selector is “OFF” and could transmit the *E. coli* gene state into the output channel if the selector could be switched “ON”, so that the LOD reaches 100 fM for both *S. aureus* and *E. coli* genes due to the effect of G-rich enhancement on the emission of DNA-AgNCs. This design could detect multiple targets with a single signal indicator [124]. A study conducted by Laibao Zheng introduced a sensing platform based on DNA-AgNCs integrated with MNPs, serving as a separation unit, DNAzyme (for bacterial recognition), and acetylcholinesterase (AChE, as the enzyme unit) for the detection of *E. coli* as the model target. When *E. coli* lysate is present, the DNAzyme reacts with target molecules released from the lysed bacteria, forming the MDA (MNP/DNAzyme/AChE)–target complex. This reaction causes the DNAzyme substrate to cleave into two fragments, releasing AChE into the solution. After magnetic separation, the liberated AChE is transferred into a system containing DNA-AgNCs. There, AChE catalyzes the hydrolysis of acetylthiocholine (ATCh) to produce thiocholine (TCh), which significantly enhances the fluorescence of the DNA-AgNCs. The LOD was obtained at 60 CFU/mL, and the recovery was in the range of 91.3–93.2% in spiked milk samples and 95–106% in spiked tap water. Notably, through modification of the DNAzyme probe, this platform can be adapted to detect a diverse range of bacterial species [125].

##### CuNCs

In a study by Sonam Kumari and colleagues, they described the creation of a highly sensitive “turn-on” fluorescent nanosensor utilizing a FRET system for the real-time detection of *E. coli*. CuNCs encapsulated within a metal–organic framework (CuNCs@ZIF-8) were synthesized as a fluorescent donor. Additionally, MnO_2_ nanospheres, known for their strong adsorption and quenching capabilities, were synthesized as the receptor. A nanocomposite (CuNCs@ZIF-8@MnO2) was designed to construct a sensing platform based on the p-benzoquinone/hydroquinone (p-BQ/HQ) redox system. In the absence of *E. coli*, fluorescence is quenched through energy transfer when MnO2 nanospheres interact with CuNCs@ZIF-8. However, upon *E. coli* exposure, NADH-quinone reductase catalyzes the reduction of p-BQ to HQ, leading to the conversion of MnO2 into Mn^2+^. This process releases the nanospheres and restores fluorescence in the composite, enabling detection. The developed FRET ON–OFF–ON fluorescent probe effectively detects *E. coli* with an LOD of 8 CFU/mL within 50 min. This sensor can detect *E. coli* bacteria in water, and the recoveries ranged from 94.3% to 106.5%. This method provides high specificity and sensitivity for identifying *E. coli* due to its dependence on the metabolic activity of the bacteria, offering significant advantages over traditional sensors [126].

##### Bimetallic or Polymetallic NCs

Sheini A. developed a rapid fluorometric assay using AuNCs and CuNCs on a paper-based platform to detect bacteria such as *S. aureus*, *Streptococcus pyogenes* (*S. pyogenes*), *E. coli*, and *Pseudomonas aeruginosa* (*P. aeruginosa*) in serum samples. The assay relies on fluorescence quenching caused by the aggregation of NCs, which is analyzed via a smartphone under UV light. A key innovation of this study is the use of six AuNCs and CuNCs stabilized with different proteins (e.g., pepsin, trypsin, ovalbumin, and glutathione), enabling selective fluorescence quenching. This allows for the differentiation of bacterial species through unique interactions between the NC compositions and bacterial cell wall structures, resulting in NC aggregation and thus the aggregation-caused quenching (ACQ) mechanism. NC aggregation is induced by hydrogen bond formation between functional groups in the protein-stabilizing agent (e.g., carboxamides thiols, carboxylic acids, and alcohols) and the active sites of the peptidoglycan present in the cell walls of bacteria. This assay was effectively used to diagnose septicemia in 40 children, highlighting its potential as a fast, affordable, and highly sensitive tool for clinical diagnostics and infection detection [127].

In another study, a fluorescent sensing array platform was developed using antibiotic-stabilized MNCs for detecting multiple pathogens. By employing five commonly used antibiotics, eight distinct NCs were synthesized, including ampicillin-CuNCs, cefepime-AuNCs and -CuNCs, kanamycin-AuNCs and -CuNCs, lysozyme-AuNCs, and vancomycin-Au/AgNCs and -CuNCs. Due to the differential interactions between each NC and bacterial strain, unique response patterns were generated. Various machine-learning algorithms were applied for pattern recognition, among which artificial neural networks demonstrated the highest performance. Moreover, due to the different affinity and interaction of each of the antibiotics against each pathogen, the emission intensity of the NCs either decreased with the ACQ mechanism or increased with the AIE mechanism, leading to a unique fingerprint for each pathogen. This work highlights the potential of using common antibiotics for NC synthesis as recognition elements in pathogen detection [128].

#### 3.3.3. Electrochemical Nanobiosensors

In recent years, NCs as an electrochemical probe have attracted interest due to their easy functionalization, large specific surface area, good conductivity, and low toxicity due to their smaller size compared to that of traditional NPs [85].

##### AuNCs

Tieu et al. demonstrated the detection of Gram-positive bacteria using electrochemical impedance spectroscopy by employing AuNCs on a gold-interdigitated wave-shaped electrode integrated with a printed circuit board (Au-PCB). Additionally, vancomycin-modified silica nanoparticles (SiNPs-VAN) were utilized to specifically bind Gram-positive bacteria, enabling their capture on the AuNC-coated surface. They reported detection limits of 102, 101, and 102 CFU/mL for *S. aureus*, *Bacillus cereus* (*B. cereus*), and *Micrococcus luteus* (*M. luteus*), respectively, within 20 min [129]. AuNCs were also used in other electrochemical approaches for pathogen detection. Wu et al. developed a signal-off electrochemical DNA sensor (E-DNA sensor) for *S. aureus* detection using a combination of a pb^2+^-specific DNAzyme and DNA walker, accompanied by Van-AuNCs and an identification aptamer strand. The system used two proximity probes: Probe 1, modified with Van-AuNCs, strongly interacted with the peptidoglycan on the Gram-positive bacterial cell wall, while Probe 2 contained an aptamer for *S. aureus*. When both probes recognized *S. aureus*, their proximity enhanced the corresponding melting temperature, enabling the formation of a Pb^2+^-dependent DNAzyme, which cleaved a track DNA into two fragments. This cleavage altered the structure of a hairpin DNA, increasing the distance between a methylene blue (MB) tag and the electrode surface, and reducing the faradaic current (signal-off). Without *S. aureus*, the hairpin structure kept MB close to the electrode, enhancing electron transfer (signal-on). This configurational change allowed the quantitative detection of *S. aureus* through redox current measurements. A range of 10–107 CFU/mL and a low LOD at 1 CFU/mL was reported [130].

##### AgNCs

AgNCs are among other MNCs used to electrochemically detect pathogenic bacteria. Ye et al. developed an electrochemical sensing strategy for detecting the *inv*A gene sequence of *Salmonella*, utilizing AgNCs as a direct signal generator and a cytosine-rich (C-rich) signal probe DNA conjugated with gold nanoparticles (sDNA-AuNPs). Target DNA and sDNA-AuNPs created a sandwich-like configuration when hybridized with a thiolated capture probe on a glassy carbon electrode. Then, with the addition of Ag^+^ and NaBH_4_, the C-rich sDNA within this structure served as a template for AgNC formation, enabling direct voltammetric signal generation during target DNA detection. The biosensor demonstrated a linear response range from 1 fM to 0.1 nM, with a detection limit of 0.162 fM [131]. Zhang et al. also employed AgNCs in an electrochemiluminescence (ECL)-based platform for *Helicobacter pylori* (*H. pylori*) DNA detection. In this study, a target-cycling synchronized rolling circle amplification (RCA) strategy was developed on a microchip. The RCA process generated amplified DNA products, which served as templates for forming DNA-stabilized AgNCs. These AgNCs catalyzed the electroreduction of potassium persulfate (K2S2O8), a co-reactant in the ECL reaction. As a result, the co-reactant near the electrode surface was efficiently consumed, allowing a significant decrease in ECL intensity. Additionally, the method was validated using clinical samples [132].

##### CuNCs

**Figure 4:**
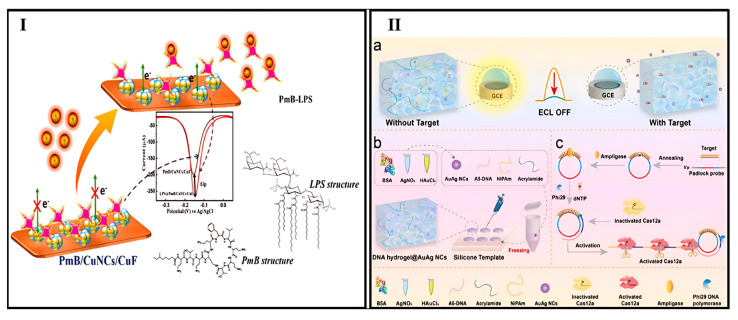
(**I**) Illustration of the LPS detection mechanism using the PmB/CuNCs/CuF sensing platform [133]. (**II**) ECL Biosensing Platform for CLas Omp Gene Utilizing RCA Amplification and CRISPR/Cas12a-Activatable DNA Hydrogel: (**a**) ECL biosensor detection mechanism; (**b**) DNA hydrogel templated synthesis of AuAg nanoclusters; (**c**) RCA-mediated target amplification triggering CRISPR/Cas12a activity [134].

CuNCs are cost-effective with high photoluminescence behavior, making them suitable alternatives for AuNCs. Thomas et al. developed blue-emitting CuNCs stabilized with cetyltrimethylammonium bromide (CTAB) for the detection of endotoxin (a bacterial toxin in Gram-negative bacteria) or LPS. In this work, at first, the CuNCs were formed on the surface of the copper foil (CuNCs/CuF). Then, with the addition of Polymyxin B (PmB) as a bioreceptor (PmB/CuNCs/CuF) and its immobilization, the differential pulse voltammetry (DPV) decreased. Finally, in the presence of LPS, the DPV signal increased through stronger interactions of PmB with LPS compared to the CuNCs [133] (Figure 4I). The efficacy of the designed sensor with an LOD of 100 ag mL^−1^ was evaluated in blood serum samples.

##### Bimetallic NCs

Bimetallic NCs as a new class of ECL emitter with an excellent quantum yield, small size, and photostability have attracted great attention. Zhang et al. reported an ECL biosensor for detection of the *Candidatus* Liberibacter asiaticus (CLas) outer membrane protein gene using Au/AgNCs. This system worked based on coupling RCA with a CRISPR/Cas12a-responsive smart DNA hydrogel containing Au/AgNCs. Without the target, the padlock probe cannot be linked to a circle to produce RCA products. Therefore, Cas12a cannot be activated, and the DNA hydrogel on the surface of GCE electrode cannot release Au/Ag NCs; therefore, the ECL signal is high. In the presence of the target, RCA produces a significant number of amplicons, which then hybridize with crRNA to activate the trans-cleavage activity of CRISPR/Cas12a. This activation triggers the smart DNA hydrogel to release encapsulated Au/AgNCs on the electrode, resulting in a reduction in the ECL signal. The change in ECL intensity showed a positive correlation with the target concentration, ranging from 50 fM to 5 nM, and achieved a detection limit of 40 fM [134] (Figure 4II). Sun et al. introduced a dual-mode sensor for LPS detection using surface-enhanced Raman spectroscopy (SERS) and ECL. The sensor leveraged poly-cytosine (poly-C) DNA to induce anisotropic etching of Au@Ag nanocubes (Au@AgNCs) in the presence of LPS, triggered by RCA. This etching altered the morphology and composition of Au@AgNCs, reducing SERS signals. Simultaneously, poly-C DNA adsorbed Ag^+^ ions, which were reduced to AgNCs for ECL signal generation. A transformation occurs from Au@Ag nanocubes to Ag NCs. The sensor achieved linear detection ranges of 1 fg mL^−1^ to 1 ng mL^−1^, with detection limits of 0.29 fg mL^−1^ (SERS) and 0.14 fg mL^−1^ (ECL) [135]. Table 1 represents an overview of current advances in NC-based biosensors for bacterial detection.

### 3.4. MNC-Based Biosensors for the Detection of Viral Pathogens

#### 3.4.1. Colorimetric-Based Optical Nanobiosensors

##### CuNCs

Mao et al. introduced DNA-templated CuNCs for the colorimetric detection of Hepatitis B virus DNA. In this system, target DNA was hybridized with a capture probe to form double-stranded DNA (dsDNA), which acted as a template for the formation of CuNCs through the reduction of CuSO_4_ by ascorbic acid (AA). Unlike other DNA-templated NCs, CuNCs formed on dsDNA exhibited high selectivity. Upon adding HNO_3_, the CuNCs were oxidized into copper ions, which then formed a copper–creatinine complex in the presence of creatinine. This complex acted as a metal mimic enzyme, catalyzing the rapid oxidation of H_2_O_2_ and ABTS to produce green-colored oxidized ABTS, detectable at 420 nm. The intensity of the color change corresponded to the amount of target DNA, enabling quantitative detection with an LOD of 12 × 10^9^ mole [136].

##### Fe_3_O_4_ NCs

**Figure 5:**
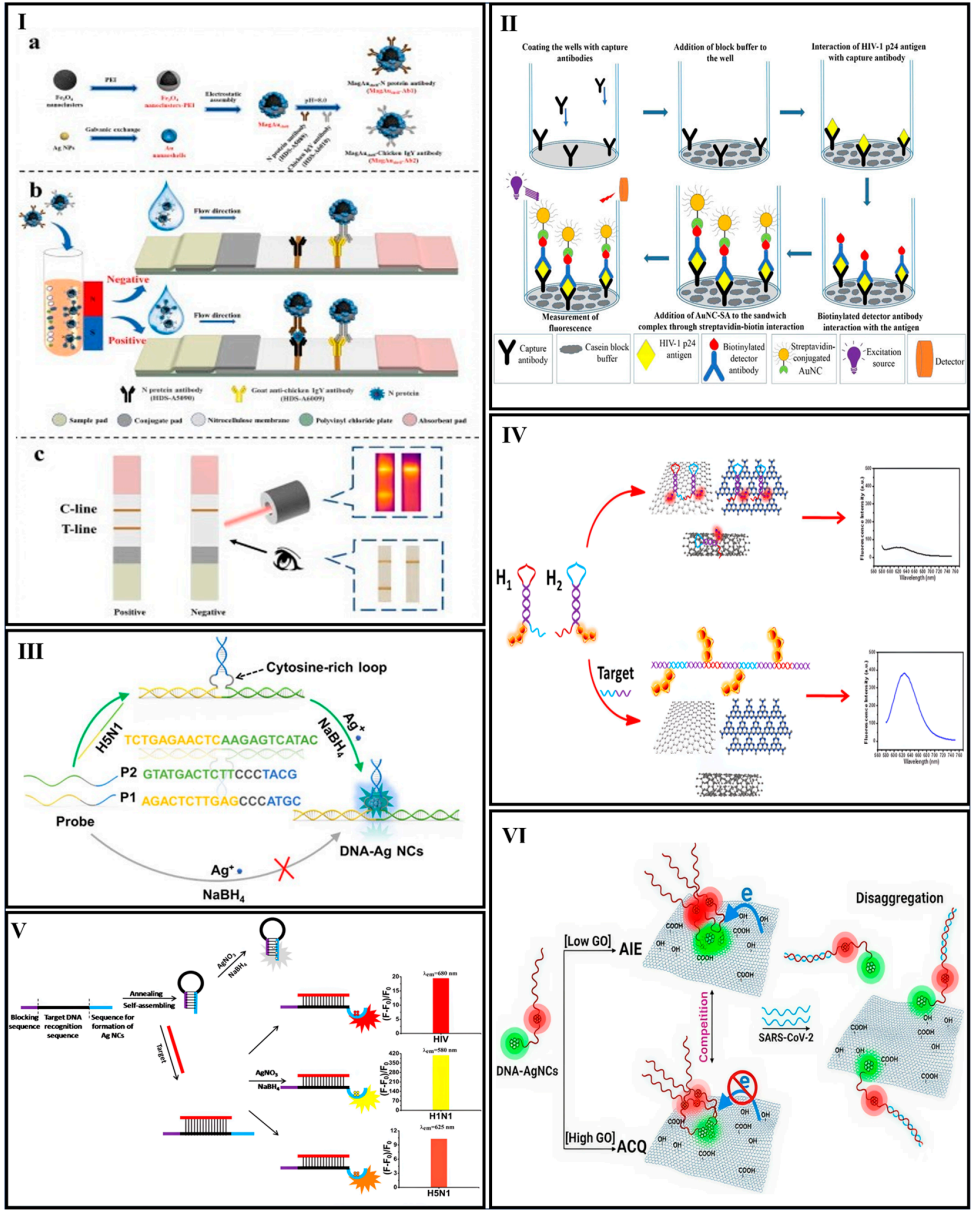
(**I**) (**a**) Synthesis and functionalization process of MagAushell NPs for biosensing applications. (**b**) Structural design and working principle of the LFIA-based biosensor for SARS-CoV-2 detection. (**c**) Interpretation of test results, including visual and infrared thermal imaging of positive and negative samples [137]. (**II**) Gold nanocluster-based immunoassay (AuNCIA) for HIV-1 p24 antigen detection [139]. (**III**) Synthesis of fluorescent DNA-templated silver nanoclusters (DNA-AgNCs) using a three-way branched DNA scaffold for H5N1 detection [142]. (**IV**) Development of multifunctional silver nanoclusters/graphene oxide (AgNCs/GO) fluorescence platform for label-free DNA detection via hybridization chain reaction (HCR) amplification [144]. (**V**) Schematic representation of target DNA detection through fluorescence signal amplification triggered by hybridization with a hairpin DNA probe [149]. (**VI**) Representation of a dual-emission ratiometric DNA-templated silver nanoclusters for label-free COVID-19 detection [93].

Fe_3_O_4_ NCs were also used for the detection of the SARS-CoV-2 nucleocapsid protein. Li et al. introduced a novel lateral flow immunoassay (LFIA) labeling system using polyethylenimine (PEI)-functionalized Fe_3_O_4_ NCs coated with high-density gold nanoshells (MagAushell). This nanoplatform enabled simultaneous bacterial enrichment and dual-mode photothermal/colorimetric detection through an integrated approach. This system used antibody-functionalized MagAushell NPs to capture and enrich the N protein from samples by magnetic separation. The captured N protein (MagAushell–Ab1–N protein complex) was loaded onto a test strip, where it formed a brown stripe on the T-line when bound by N protein antibodies. A second brown stripe appeared on the C-line as a control, since the MagAushell–Ab2 with the chicken IgY antibody continued to flow to the C-line area and was captured by the goat anti-chicken IgY antibody on the C-line. For negative samples without N protein, only the C-line showed a stripe, while no stripe on the C-line indicated an invalid test. Qualitative detection was achieved by visually observing the color stripes, while quantitative analysis was performed by measuring temperature changes under laser irradiation, enabling dual-mode detection without the need for complex instruments [137] (Figure 5I).

##### Magnetic NCs

Magnetic NCs were also used for the detection of African swine fever virus (ASFV). In a study conducted by Lee et al., an approach leveraging an on-particle hairpin chain reaction to functionalize magnetic NCs with long DNA strands in a target-dependent fashion was introduced. These NCs were then introduced into a column chromatography device, producing signals that were both quantifiable and visible to the naked eye. The system detected five genes of the ASFV genome with a sensitivity of ≈19.8 pM in swine serum, completing the process in 30 min at room temperature. By incorporating a PCR pre-amplification step, the assay accurately identified ASFV in 30 suspected swine samples, matching the 100% sensitivity and specificity of quantitative PCR [138].

#### 3.4.2. Fluorescence-Based Optical Nanobiosensors

##### AuNCs

AuNC-based biosensors have been developed to detect various viral targets, including Human Papillomavirus (HPV) and Human Immunodeficiency Virus (HIV), with high sensitivity and specificity. Kurdekar et al. developed an AuNC immunoassay (AuNCIA) using streptavidin protein-labelled fluorescent glutathione-AuNCs for early and sensitive HIV detection. AuNCIA demonstrated threefold higher sensitivity and specificity than ELISA, showing 100% specificity against non-HIV samples, including those positive for hepatitis B, hepatitis C, and dengue, highlighting its potential for universal pathogen detection. In this work, after coating microtiter wells with the capture antibody, the HIV-1p4 antigen was added; after the antibody–antigen interaction, a sandwich complex was formed with the secondary biotinylated anti-p4 antibody. The immunocomplex was attached to the AuNC-SA via streptavidin/biotin interaction, and the fluorescence signal was measured at 615 nm under an excitation of 345 nm. The enhancement effect of AuNCs for binding to the protein compared to other MNCs was evaluated by silico studies. Moreover, the LOD of 5 pg mL^−1^ was achieved in 100 clinical samples (i.e., 50 HIV-negative and 50 HIV-positive samples) [139] (Figure 5II). In another study, Chellasamy et al. developed a dual-functional sensor based on the efficient electrocatalytic activity of AuNCs for SARS-CoV-2 nucleoprotein detection. The density functional theory (DFT) method was also used to analyze the electronic structure of gold (III) chloride and BSA interactions for AuNCs’ formation. This study combined fluorescence and electrochemical sensing, with antibodies conjugated to AuNCs for improved antigen binding. The detection limit was found to be about 20 pM with high responsiveness and specificity toward SARS-CoV-2 nucleoprotein detection [140].

##### AgNCs

In their study, Cao et al. developed an AgNC-based fluorescence biosensor for detecting the genetic sequences of HIV, HBV, and human T-lymphotropic virus 1 (HTLV-I). The fluorescence intensity of DNA-AgNCs decreased upon hybridization with target DNA molecules, leading to a sensitive detection method. The DNA probe included a cytosine-rich sequence for the synthesis of AgNCs, a G-rich sequence as a signal reporter, and a loop structure of the complementary sequence to target DNA. In the absence of target DNA, the DNA-AgNC probe had a hairpin structure, allowing the fluorescence enhancement of AgNCs due to its proximity to the GRS. After spiking target DNA, the hairpin loop opens and induces AgNCs away from the GRS, leading to fluorescence quenching. The LOD for HIV, HBV, and HTLV-I was reported as 4.4 nM, 6.8 nM, and 8.5 nM, respectively [141]. Another study by Zhang et al. introduced a label-free fluorescence biosensing platform based on DNA-templated AgNCs for detecting the Avian Influenza A (H5N1) virus gene sequence. By employing a three-segment-branched DNA structure with a cytosine-rich loop and two P1 and P2, they achieved a wide linear detection range (500 pM–2 μM) and a low LOD of 500 pM. In the presence of H5N1, P1 and P2 complementary DNA probes hybridize with the H5N1 target but not completely, and thus a three-segment-branched DNA structure with a closed cytosine-rich loop is formed. With the addition of AgNO_3_ and NaBH_4_, fluorescence at 500 nm could be produced by AgNC formation in the loop structure [142] (Figure 5III). Additionally, Shen et al. developed a DNA-AgNC probe with a GCC-loop structure. In the presence of target DNA, the secondary structure of the DNA probe changes due to its hybridization with the target DNA. This allows the release of the GCC-loop as an NC-stabilizing template. After that, AgNCs can be synthesized with high emission by adding NaBH_4_ and Ag+. Therefore, a “turn-on” fluorescence probe was designed for Norovirus RNA with a linearity ranging from 0.02 μM to 1.8 μM with an LOD of 18 nM. Moreover, the addition of a double-stranded structure to the GCC-loop significantly enhanced fluorescence intensity [143]. Zhang et al. also developed a fluorescence biosensor for HIV detection using DNA-AgNCs/GO and the hybridization chain reaction (HCR). In the absence of target DNA (THIV), two hairpin DNA-AgNCs probes (H1, H2) with partially complementary sequences were captured on the surface of GO, and thus fluorescence quenching occurred. Therefore, HCR could not be induced due to the stability of H1-AgNC and H2-AgNC probes on the GO surface, leading to a low signal background. By adding THIV, the chain-like assembly of H1 and H2 was triggered through HCR, producing AgNCs nanowires or a long chain of H1-AgNC and H2-AgNC complexes. The obtained HCR product could not be adsorbed on the GO surface, and thus a strong emission was generated at 623 nm based on the THIV concentration (turn-on). The LOD was 1.18 nM, and a good recovery was achieved in human serum samples [144] (Figure 5IV). Moreover, Yang et al. developed a label-free fluorescence biosensor for HIV DNA detection using luminescent DNA-scaffolded AgNCs and exonuclease III-assisted signal amplification. The system operates in a single step without DNA labeling, where exonuclease digestion triggers fluorescence quenching [145]. In addition, Chen et al. developed a fluorescence polarization (FP)-based biosensor for the detection of HBV DNA using SiO_2_ NP–DNA/AgNCs as a sensing platform. In this work, at first the biotin-En-DNA probe was immobilized on streptavidin-SiO_2_ NPs. Then the Ag-DNA probe was added, and a small emission could be produced in the absence of the target. Finally, in the presence of HBV target DNA, a sandwich structure was formed on the surface of SiO_2_ NPs, and a strong signal was recorded (turn-on) [146]. In another study, Yuan et al. utilized C-rich DNA templates to create the fluorescent Probe 1-AgNCs and non-fluorescent Probe 2-AgNCs, which emitted bright yellow and weak red fluorescence, respectively. Upon the hybridization of HPV-16 DNA with Probe 1-AgNCs and Probe 2-AgNCs, the fluorescence color of AgNCs shifted from yellow to red, forming a nanocluster dimer (NCD) and enabling the ratio-based detection of HPV. This method was able to detect HPV in human serum, with an LOD of 2 nM [147]. In a study by Zou et al., a novel AgNC-based label-free fluorescent platform was developed for simultaneously detecting two HIV oligonucleotides in human serum samples based on the guanine-rich enhancement method. Green (G-AgNCs) and red (R-AgNCs) fluorescent DNA-AgNCs were formed using different DNA templates, emitting at 565 nm and 630 nm, respectively. In the presence of HIV-1 or HIV-2 DNA, the guanine (G)-rich overhang was displaced, leading to fluorescence intensity reduction. The platform demonstrated high selectivity for multiplexed DNA analysis with a detection limit of 11 pM [148]. Han et al. also developed a fluorescent molecular beacon using DNA-AgNCs for detecting multiple virulence genes. The designed beacon with three segments (blocking, recognition, and AgNC template sequences) allowed the simultaneous detection of different nucleic acid targets due to the distinct emission spectra of synthesized AgNCs. Based on target-induced fluorescence enhancement, the system detected H1N1, H5N1, and HIV genes with detection limits of 0.12, 3.95, and 3.53 nM, respectively [149] (Figure 5V). Similarly, Wu et al. developed a novel AgNC-based fluorescence probe for HIV/ hepatitis C virus (HCV) DNA detection by integrating Exo III-assisted target recycling amplification with rolling circle amplification. The designed padlock probes contained a G-rich region, and target DNA triggered Exo III digestion, releasing numerous ssDNA fragments that initiated RCA. This process generated cytosine-rich (C-rich) units, leading to in situ AgNC formation and strong fluorescence. The assay achieved a detection limit of 1.4 fM and could differentiate multiple viral DNAs using AND/OR logic circuits [150]. In another study, Qu et al. demonstrated a fluorescence-based sensing strategy using polymer-capped AgNCs and MoS2 nanosheets for detecting HBV DNA. PEI-AgNC fluorescence was quenched by MoS2 nanosheets but restored upon nanosheet aggregation in the presence of sodium acetate (50 mM). However, with the addition of the ssDNA target, its adsorption happens on MoS2 nanosheets, leading to re-quenching fluorescence. The addition of complementary DNA or an aptamer substrate reversed this effect, restoring fluorescence (on–off–on fluorescence detection) [151]. In a study, Ye et al. developed a novel FRET-based sensor for detecting HIV DNA using DNA-AgNCs and carbon nanoparticle (CNP) oxide based upon a dual-probe strategy (P1 and P2) to enhance sensitivity. In the absence of target DNA, the fluorescence of AgNCs was quenched by CNP oxide by the FRET mechanism. In the presence of target DNA, a dsDNA hybrid formed, leading to the desorption of the ssDNA-AgNCs probes from CNP oxide surface, reducing FRET and hence the fluorescence recovery (off–on). The sensor detected HIV DNA in the range of 1–50 nM with a detection limit of 0.40 nM [46]. In another study, Molaabasi and her colleagues developed a rapid, ratiometric fluorescent biosensor for COVID-19 diagnosis that operates without labels, antibodies, enzymes, or amplification steps, delivering results from isolated RNA in under 12 min. The biosensor, based on cytosine-modified antisense oligonucleotides targeting the N gene of SARS-CoV-2 or the RDRP gene, forms AgNCs with both green and red emissions. The presence of target RNA generates a dual-emission ratiometric signal, achieving a 0.30 to 10.0 nM limit of detection, without requiring further amplification, special DNA fragments or fluorophores. Moreover, the effects of the T/C ratio, the number of cytosines in the loop and in the 3′ end of the AgNC-stabilizing template, and the stem length on the sensitivity of sensing were investigated. Importantly, the effect of graphene oxide (GO) on the ratiometric behavior of NCs was investigated and the time-/concentration-based competitive mechanism between ACQ and AIE for the ssDNA-AgNCs/GO nanohybrids was demonstrated, so that in the presence of the RNA of SARC-CoV-2, disaggregation happened and the ratiometric behavior of the green- and red-emitting clusters was reversed [93] (Figure 5VI). Lv et al. developed a method for HBV DNA detection using a concentration imbalance-driven DNA circuit (CIDDC) as an amplifier, coupled with a DNA-AgNCs nanoprobe. The CIDDC system amplifies HBV DNA, triggering fluorescence from the AgNC reporter. This approach achieved high sensitivity with a detection limit of 0.11 nM and single-base mismatch discriminability, offering a simple, isothermal, and user-friendly assay for HBV DNA detection [152]. Fang et al. developed a fluorescent DNA-AgNCs probe for HIV DNA detection. DNA containing six cytosines was used to form AgNCs, and it was discovered that the fluorescence of DNA-AgNCs could be quenched after hybridization with a complementary sequence. By designing a sequence (C1) complementary to HIV DNA, the fluorescence was turned “on” after HIV DNA induced a strand exchange reaction, releasing C1. This “off–on” fluorescence method allowed HIV DNA detection with a limit of detection of 3.18 nM, demonstrating excellent specificity, even for mismatched HIV DNA [153]. Off–on fluorescence detection was also applied by Wang et al. based on a FRET mechanism between complementary DNA–AgNCs and multi-walled carbon nanotubes (MWCNTs) as quenchers, so that in the presence of respiratory syncytial virus (RSV) gene sequences and its hybridization with complementary DNA-AgNCs, the fluorescence of AgNCs was enhanced as a result of DNA-AgNC desorption from the MWCNT surface. Using DNA–AgNCs and MWCNTs, the sensor achieved a high quenching efficiency (85.8%) and a detection limit of 24 nM. The fluorescence signal increased linearly with RSV DNA concentration from 31.25 nM to 2.00 μM, offering a sensitive detection method [154].

Multiplexed virus detection using AgNCs has also been achieved through hybrid systems. Liu et al. developed a biosensing platform combining AgNCs with GO for simultaneous detection of HIV and HBV genes. The fluorescence of AgNCs was quenched upon binding to GO, and upon interaction with target viral genes, fluorescence was restored (off–on), enabling dual-target detection with LODs of 1 nM for HIV and 0.5 nM for HBV [155].

##### CuNCs

DNA-stabilized CuNCs have attracted much attention with respect to their low cost, excellent optical features, preferable biocompatibility, large stokes shifts, and fast and easy synthesis under ambient conditions. All of these show that CuNCs can act as powerful complements to organic fluorophores or fluorescent proteins in bioanalysis. Du et al. developed a CuNC-based assay for the detection of the SARS-CoV-2 Delta variant, distinguishing it from the wild-type strain using rationally designed primers. Dual priming oligonucleotide (DPO) primers and AT primers enabled specificity in PCR analysis, while red fluorescence from CuNCs under UV light provided a visual readout. In fact, the AT primers enhanced the AT contents of PCR products, thereby giving the template concentration needed to generate CuNCs. A linear range from 0.5 pg μL^−1^ to 50 ng μL^−1^ and a limit of quantitation of 0.5 pg μL^−1^ were obtained [156]. Tao et al. developed a “turn-off” fluorescence biosensor for HBV detection using CRISPR-Cas12a and DNA-MNCs (AuNCs, AgNCs, and CuNCs), achieving picomolar sensitivity within 25 min. Among these, CuNC-based biosensors exhibited the highest sensitivity and fastest fluorescence response, with an LOD of 0.54 pM, providing a rapid and efficient detection platform. In the absence of a DNA target, highly fluorescent MNCs could be synthesized by DNA probes, while in the presence of the HBV target the trans-cleavage capability of Cas12a was able to degrade DNA probes, which inhibited MNC formation, leading to minimal fluorescence signals (on–off) [35]. In another study, Dhanasekaran et al. developed an immunosensor for detecting monkeypox (MPXV) by synthesizing red-fluorescent CuNCs. In this regard, both fluorescence and electrochemical sensing were performed in PBS, achieving detection limits of 0.096 nM and 0.114 nM, respectively. X-ray photoelectron spectroscopy analysis revealed the antigen–antibody interaction mechanisms, and the immunosensor demonstrated successful detection of the A29P antigen in spiked serum samples using fluorescence, smartphone colorimetric, and electrochemical sensing techniques [157]. In addition, Shokri et al. developed a competitive fluorescence anisotropy immunoassay for Citrus Tristeza Virus (CTV) detection by specifically targeting the viral capsid protein CP25. CP25, which was expressed in *E. coli*, was used to synthesize fluorescent CuNCs with emission in the range of 535–715 nm. The CP25-CuNCs interacted with the anti-CTV antibody, and the fluorescence anisotropy values decreased linearly from 400 pg/mL to 25 ng/mL with increasing CP25 protein levels, achieving an LOD of 220 pg/mL [32]. Chen et al. developed a sensitive ratiometric fluorescence biosensor for HTLV-I DNA detection using in situ-generated CuNCs on DNA-modified graphene quantum dots (GQDs). Exonuclease III-assisted target recycling released AT-rich single-stranded DNA, which hybridized with complementary DNA1 on GQDs, serving as a template for CuNC formation (off–on). In the absence of target DNA, exonuclease I degraded DNA1, preventing CuNC generation. With GQDs as a reference and CuNCs as a reporter, the assay exhibited a detection limit of 10 pM and effectively detected HTLV-I DNA in human serum [158]. Zheng et al. developed an approach for the rapid detection of HCV, combining a 3D DNA walking nanomachine with catalytic hairpin assembly (CHA) and CuNCs. The integration of CHA with the 3D DNA walking nanomachine enhanced target amplification, leading to faster reactions and improved detection performance. Terminal deoxynucleotidyl transferase (TdT) was used to generate T-rich DNA sequences, which served as templates for CuNC formation, providing the detection signal. The biosensor achieved high sensitivity with a detection limit of 42.4 pM and a linear range from 100 pM to 2 nM [159].

#### 3.4.3. Electrochemical Nanobiosensors

##### AuNCs

**Figure 6:**
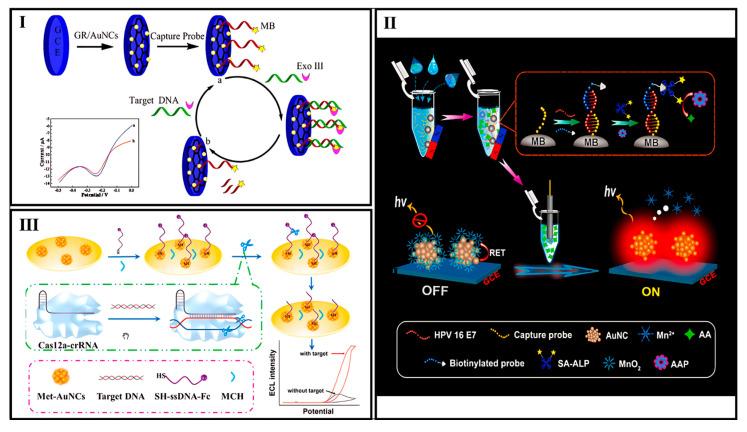
(**I**) Schematic representation of the biosensor fabrication process: (a) initial signal generated by the capture probe; (b) final signal after target DNA incubation with Exo III [160]. (**II**) Schematic overview of the split-type ECL DNA biosensor using AuNCs for HPV16 E7 detection [161]. (**III**) Diagram illustrating the working principle of the Cas12a-based ECL biosensor for HPV-16 DNA detection [162].

Electrochemical approaches have been developed for detecting viruses based on fluorescent MNCs. Wang et al. reported using graphene-stabilized gold nanoclusters (GR/AuNCs) for the detection of HIV with DPV. In this approach, a GR/AuNC-modified glassy carbon electrode (GCE) platform with exonuclease III (Exo III)-assisted DNA recycling amplification based on a C-rich aptamer was used. Aptamers were labeled with MB at the 3′ end and a C-rich sequence at the 5′ end as a capture probe for target DNA detection. The C-rich probe bound efficiently to the GR/AuNCs platform, producing a clear MB signal. When target DNA was present, the probe formed a duplex structure, enabling Exo III to digest the probe from the 3′ end, releasing MB, and recycling the target DNA, which resulted in a DPV signal decrease, acting as a signal-off electrical sensor. The biosensor with an LOD of 30 aM could be used for target analysis in human serum, while the obtained recoveries were between 93.4 and 99.8% [160] (Figure 6I). Hong et al. developed an AuNC probe-based split-type ECL assay platform for HPV detection. A “sandwich” hybridization approach was used, where amino-functionalized capture probes were covalently attached to the surface of magnetic beads for easy magnetic purification and separation. When HPV16 E7 and a biotin-labeled reporter probe were present, a sandwich structure formed by hybridization. After the biotin/avidin reaction between streptavidin-alkaline phosphatase (SA-ALP) and biotinylated DNA complex, AA was produced from L-ascorbic acid 2-phosphate trisodium salt (AAP). Finally, the modified electrode MnO_2_/AuNCs/GCE was immersed in the purified AA solution. Then the ECL signal, which decreased due to resonant energy transfer (ECL-RET) between AuNCs and MnO_2_ nanosheets, recovered significantly after MnO_2_ etching by AA, enabling sensitive HPV16 E7 detection. Here, the reduced AuNCs served as high-performance ECL probes, showing strong and stable signals. The use of magnetic beads enhanced sensitivity and anti-interference capabilities, while the split-type design improved efficiency by separating nucleic acid hybridization and ECL detection, avoiding steric hindrance and sample complexity issues. The developed design was evaluated for 27 clinical samples with a good Pearson’s correlation (R = 987) [161] (Figure 6II). In a study by Liu et al., a Cas12a-based ECL biosensor for amplification-free DNA detection was developed, leveraging Cas12a’s dual recognition mechanism for specificity and its trans-cleavage activity for signal amplification. L-Methionine-stabilized gold nanoclusters (Met-AuNCs) served as efficient ECL emitters. The biosensor targeted HPV-16 DNA. Initially, Met-AuNCs on the electrode provided an ECL signal, which was quenched by ferrocene-tagged thiolated ssDNA (SH-ssDNA-Fc). In the presence of HPV-16 DNA, Cas12a was activated, cleaving SH-ssDNA-Fc and restoring the ECL signal. The biosensor achieved a detection limit of 0.48 pM, outperforming most amplification-free CRISPR-based methods, and demonstrated reliable detection in undiluted human blood samples. Its programmable crRNA design allows adaptation for detecting other DNA biomarkers, making it a versatile and promising platform for CRISPR/Cas-based ECL biosensors [162] (Figure 6III).

##### Palladium Nanoclusters (PdNCs)

PdNCs have been known as a catalytic reagent in electrochemical reactions such as the oxygen reduction reaction (ORR) or the hydrogen evolution reaction (HER) that occurs at a lower negative potential. In a study conducted by R. Penedo et al., PdNCs were used as endpoint indicators for the detection of SARS-CoV-2 carried out by reverse transcription loop-mediated isothermal amplification (RT-LAMP). As the reaction progressed, the pH of the medium decreased, leading to a measurable change in the catalytic activity of PdNCs toward the ORR, which was detected electrochemically (voltammetry). The detection process utilized small-sized screen-printed electrodes pre-modified with PdNCs, combined with compact and straightforward electrochemical instrumentation. A concentration of 100 copies µL^−1^ of the fragment N1 of SARS-CoV-2 was considered the practical LOD of the methodology [163]. Table 2 represents an overview of current advances in NC-based biosensors for virus detection.

## 4. Challenges

MNCs have emerged as promising candidates for the detection of bacterial and viral targets, demonstrating varying levels of efficacy depending on their composition and properties. Among the various types of MNCs, AuNCs, AgNCs, and CuNCs are the most extensively reported in the literature, owing to their distinct physicochemical properties that significantly influence their performance in optical and electrochemical biosensing platforms. AuNCs exhibit outstanding chemical stability and size-dependent photoluminescence. Their excellent biocompatibility and ease of surface functionalization make them highly suitable for use in both optical and electrochemical biosensing applications [59]. Similarly, AgNCs exhibit a distinctive combination of physicochemical characteristics, including bright fluorescence, as well as favorable electronic, catalytic, and antibacterial properties. Together with their simple and tunable synthesis, these attributes render AgNCs highly advantageous for the development of sensitive and selective biosensing platforms [164]. In colorimetric biosensing applications, AuNCs and AgNCs demonstrate enzyme-like catalytic activities, such as peroxidase-mimetic functions, which facilitate observable colorimetric changes upon binding with specific analytes [50]. In contrast to gold and silver nanoclusters, copper is a more affordable, widely available, and naturally abundant element, which enhances the appeal of CuNCs for sensor development. Nevertheless, CuNCs continue to encounter significant challenges, including a low fluorescence quantum yield and a high propensity for oxidation [165].

According to the current review, MNCs have been predominantly employed in the development of fluorescence-based biosensors. In contrast, their application in colorimetric and electrochemical assays—particularly for viral detection—remains limited, revealing a notable gap in the current research regarding the broader diagnostic potential of MNCs. In addition, AgNCs have emerged as the most commonly used metal nanoclusters in fluorescence-based biosensing platforms for viral pathogen detection. In contrast, AuNCs have been more frequently utilized in biosensors designed for bacterial detection. In the majority of studies employing AgNCs for the detection of bacterial or viral targets, the nanoclusters have been synthesized using DNA scaffolds as stabilizing and templating agents. The synthesis of DNA-protected AgNCs is relatively straightforward. These DNA-stabilized nanoclusters are capable of producing ultra-bright fluorescence, making them competitive with conventional high-intensity fluorophores [34]. Nucleic acids act as stable templates for the synthesis of AgNCs, as their inherent structural and chemical characteristics contribute to the enhanced functionality of the resulting nanoclusters [165]. In contrast, AuNCs have more commonly been synthesized on protein- or peptide-based scaffolds, owing to their favorable interaction with thiol and amine groups and their enhanced biocompatibility. In many cases, the precise structures of MNCs remain poorly understood. Understanding their structure is crucial, as it can directly influence key properties such as optical and electronic behavior, which in turn can affect their performance and reliability in biosensing and other biomedical applications. Another important aspect to consider is the validation of biosensors using real biological samples. Although most reported biosensors have been tested under such conditions, some lack this critical evaluation. Real-sample testing is essential to assess biosensor performance in complex biological environments and to ensure their reliability for practical diagnostic applications.

Despite the promising results in laboratory settings, several challenges hinder the translation of MNC-based biosensors into commercially viable products. One major hurdle is the scalability and reproducibility of MNC synthesis while maintaining consistent physicochemical properties. Additionally, the stability and shelf life of these nanomaterials under various environmental conditions must be ensured. Regulatory approval processes, biocompatibility concerns, and the integration of nanomaterials into user-friendly platforms also pose significant obstacles. Moreover, cost-effective mass production and compatibility with existing diagnostic infrastructures remain unresolved issues. Finally, the multiplex pathogen detection using MNCs is limited, and the development of MNC probes with dual-modal assay capability is insufficient. Addressing these limitations through interdisciplinary collaboration, advanced materials engineering, improved encapsulation strategies, the simultaneous use of multiple probes, the design of multicolor MNCs, and the implementation of machine-learning algorithms is essential for the commercial realization of MNC-enabled biosensors. Also, most biosensors are developed using simple, cost-effective, and easily miniaturizable transduction platforms, making them suitable for clinical translation and large-scale commercialization. These systems are typically designed to offer rapid, sensitive, and user-friendly diagnostics. Nevertheless, despite their innovative concepts and theoretical potential, many biosensors remain complex in terms of design or operation. This complexity often limits their practical applicability and hinders their adoption as viable alternatives to well-established technologies.

## 5. Role of Machine Learning

The integration of machine-learning (ML) algorithms into biosensing workflows presents a transformative opportunity to enhance signal analysis and interpretation, particularly in complex or multiplexed detection scenarios. ML techniques such as support vector machine (SVM), random forest, and deep neural networks can be trained to recognize subtle signal patterns and distinguish between overlapping responses in multiplex assays. Additionally, ML can assist in optimizing biosensor calibration, compensating for background noise, and improving the robustness of readouts across variable sample conditions. This convergence of nanotechnology and data science may significantly improve the analytical power and reliability of MNC-based biosensors, especially in decentralized or point-of-care applications [105].

## 6. Conclusions and Future Perspectives

There are acceptable reports of pathogen detection based on MNCs, including HIV, HBV, HTLV-I, H5N1, Norovirus, SARS-CoV-2, Influenza, HPV-16, H1N1, H5N1, HCV, RSV, Monkeypox, CTV, HTLV-I, ASFV, and HPV as viral pathogens and *S. typhimurium*, *S. aureus*, *S. pyogenes*, *E. coli*, *P. aeruginosa*, *L. monocytogenes*, *E. coli* O157:H7, MRSA, *azospirillum baldaniorum*, lipopolysaccharide, and endotoxin for pathogenic bacteria. MNCs are capable of catalyzing the substrate, leading to a detectable color change visible to the naked eye, enhancing the electron transfer in the electrochemical system, and producing strong photoluminescence whereby MNCs can act as a photoluminescent, photoelectroluminescent, photocatalyst, and photoelectrocatalyst. Despite these advantages of MNCs, several important challenges should be considered as follows: (i) There is no precise prediction between NCs’ properties and their responsibility for pathogen detection. This can be achieved by employing machine-learning algorithms. (ii) Furthermore, most MNC probes used in colorimetric assays are related to a single metal (Au, Ag, Pt, and Cu), while greater accuracy and sensitivity may be obtained by polymetallic cores or advanced nanocomposites. (iii) The colorimetric and electrochemical assays are detection methods that have been less highlighted in using MNCs, which shows the limitation of existing projects for viral and bacterial disease diagnostics. (iv) The number of studies focusing on MNC-based virus detection is limited compared to bacterial detection, which shows it is an overlooked area of research. (v) Recently, simultaneous pathogen detection has been very interesting, while a limited number of studies have been focused on this. In this regard, designing multi-color MNCs or using multiple probes simultaneously can be proposed. (vi) Additionally, there are not many reports on the mechanism of MNC-based pathogen detection. More related studies will help to better design MNC probes. (vii) Research on stability in storage and transport still remains to be performed and also limits the practical applications of MNCs. (viii) Scalability issues are another challenge due to the high cost of noble metals (e.g., Ag, Au, and Pt), resulting in an expensive production cost. These constraints indicate that more effort is needed to research more stable, cost-effective, and widely applicable MNCs. However, this review has certain limitations. The inclusion was restricted to English-language publications, potentially introducing language bias. To maintain scientific rigor, non-peer-reviewed sources such as case reports and conference abstracts were excluded. Additionally, studies with insufficient data quality, undefined performance metrics, irrelevant biological targets, or only indirect relevance to nanocluster-based biosensing were omitted. While these criteria ensured consistency and reliability, they may have led to the exclusion of some valuable insights. In addition, based on the inclusion of high-quality, peer-reviewed studies with largely positive and consistent findings, the certainty of the evidence supporting the use of nanocluster-based biosensors for bacterial and viral detection is considered moderate to high. However, variability in methodologies across studies slightly limits our overall confidence in the results.

## Figures and Tables

**Table 1:** Nanocluster-based biosensors for bacteria detection.

Biosensing Format	Analyte (Bacteria)	Nanocluster	Linear Range	LOD	Real Sample	Ref.
Fluorescence	*S. typhimurium*	DNA-AgNCs	10^2^–10^7^ CFU/mL	50 CFU/mL	Chicken meat	[120]
	*S. typhimurium*	DNA-AgNCs	10–5.0 × 10^5^ CFU/mL	8 CFU/mL	Milk	[121]
	*S. aureus* *S. pyogenes* *E. coli* *P. aeruginosa*	Ovalbumin, pepsin, trypsin, glutathione-AuNCs and -CuNCs	50.0–1.0 × 10^8^ CFU/mL 70.0–1.0 × 10^8^ CFU/mL 30.0–1.0 × 10^8^ CFU/mL 50.0–1.0 × 10^8^ CFU/mL	43 CFU/mL 63.5 CFU/mL 26 CFU/mL 47 CFU/mL	Human serum	[127]
	*E. coli* J96	Mannose-AuNCs	-	~2 × 10^6^ cells/mL	Urine	[112]
	*S. aureus*	BSA-AuNCs/ antiSAIgG–PS complexes	-	-	-	[113]
	*L. monocytogenes*	MPA-AuNCs	2 × 10^5^–10^6^ CFU/mL	2 × 10^5^ CFU/mL	Milk	[114]
	*E. coli* O157:H7	GSH-AuNCs@chitosan nanocapsules	3–700 CFU/mL	1 CFU/mL	Drinking water, milk	[115]
	*S. aureus*	Vancomycin-AuNCs	32–10^8^ CFU/mL	16 CFU/mL	Milk, human serum	[116]
	*S. aureus* and MRSA	HSA-AuNCs	-	4.2 × 10^6^ cells/mL	Urine	[117]
	*S. aureus*	Vancomycin-AuNCs	20–10^8^ CFU/mL	10 CFU/mL	Milk, orange juice, human serum	[118]
	*E. coli* O157:H7	BSA-AgNCs- Aptamer and MBs	10–10^6^ CFU/mL	0.2549 CFU/mL and 0.6031 CFU/mL (in the milk)	Milk	[122]
	*S. typhimurium*	DNA-AgNCs	3.7 × 10^2^–3.7 × 10^5^ CFU/mL	98 CFU/mL	Milk	[123]
	*E. coli* *S. aureus*	DNA-AgNCs	-	100 fM	Food	[124]
	*E. coli*	DNA-AgNCs	1 × 10^2^–1 × 10^7^ CFU/mL	60 CFU/mL	Tap water, milk	[125]
	*E. coli* *P. aeruginosa* *S. typhimurium* *S. aureus*	Amp-CuNCs, Cef-AuNCs, Cef-CuNCs, Kan-AuNCs, Kan-CuNCs, Lys-AuNCs, Van-BMNCs, Van-CuNCs	50–1 × 10^8^ CFU/mL 10^2^–1 × 10^8^ CFU/mL 50–1 × 10^8^ CFU/mL 10–1 × 10^8^ CFU/mL	19.0 CFU/mL 43.0 CFU/mL 17.0 CFU/mL 4.5 CFU/mL	Drinking water, tap water, water from the Anzali Lagoon	[128]
	*E. coli*	BSA-CuNCs@MOF ZIF-8	5 × 10^1^ to 5 × 10^5^ CFU/mL	8 CFU/mL	Tap water, handpump water, canal water, pond water, sewage water	[126]
	*Azospirillum baldaniorum*	GSH-AuNCs.	2.6 × 10^5^ to 6.7 × 10^7^ CFU/mL	1.7 × 10^5^ CFU/mL	Urinary catheters	[119]
Colorimetry (TMB, H_2_O_2_)	*S. typhimurium*	BSA-AuNCs@aptamer	10^1^–10^6^ CFU/mL	1 CFU/mL	Eggshell, egg white	[103]
Colorimetry (TMB, H_2_O_2_)	*S. aureus/Staphylococcal enterotoxin B*	DNA-AuNCs@chitosan composite	-/1–700 ng/mL	4 × 10^2^ CFU/mL/1.0 × 10^−12^ g/mL	Food samples	[104]
Colorimetry (TMB, H_2_O_2_)	*E coli* O157:H7	Papainp-AuNCs @aptamer	10^2^–10^6^ CFU/mL	39 CFU/mL	Ultra-high temp sterilized, pasteurized, and raw milk	[105]
Colorimetry (TMB, H_2_O_2_)	*S. aureus*	GSH-AuNCs@liposomes		0.028 pmol	Mouse urine samples	[106]
Colorimetry (TMB, H_2_O_2_)	*C. jejuni* DNA	DNA-Au/Pt NCs	50 pM–100 nM	20 pM	Milk samples	[109]
Colorimetry (TMB, H_2_O_2_)	*S. aureus*	DNA-Au/Pt NCs	10^8^–10^2^ CFU/mL	80 CFU/mL	Food samples	[110]
Colorimetry (TMB, H_2_O_2_)	*S. typhimurium*	DNA-Ag/PtNCs @Aptamers-magnetic beads	2.6 × 10^2^–2.6 × 10^6^ CFU/mL	2.6 × 10^2^ CFU/mL	Milk samples	[111]
Colorimetry (OPD)	*L. monocytogenes*	IgY-AgNCs@Aptamers magnetic beads	10–10^6^ CFU/mL	10 CFU/mL	Food samples	[107]
Colorimetry (TMB, H_2_O_2_)	*S. typhimurium*	IMONCs@IMNPs	1.5 × 10^1^–1.5 × 10^6^ CFU/mL	101 CFU/mL	Food samples	[108]
Electrochemistry (impedance spectroscopy)	*S. aureus* *B. cereus* *M. luteus*	AuNCs@vancomycin-SiNPs	10^2^–10^5^ CFU/mL	10^2^/10^1^/10^2^ CFU/mL	Water samples	[129]
Electrochemistry (DPV)	*S. aureus*	Aptamer@vancomycin-AuNCs@DNA walker@pb^2+^-DNAzyme	10–10^7^ CFU/mL	1 CFU/mL	Raw milk, beer, and apple juice samples	[130]
Electrochemistry (DPV)	*invA* gene sequence of *Salmonella*	AgNCs@sDNA-AuNPs	1 fM–0.1 nM	0.162 fM	-	[131]
Electrochemiluminescence (ECL)	*H. pylori* DNA	circle DNA products-AgNCs		10 pM	Clinical samples	[132]
Electrochemiluminescence (ECL)	*Candidatus* Liberibacter asiaticus (*C*las) outer membrane protein (Omp) gene	Au/AgNCs encapsulated CRISPR/Cas12a-hydrogel	50 fM–5 nM	40 fM	Live citrus leaves	[134]
Dual-mode SERS + ECL	Lipopolysaccharide (LPS)	Cyclic poly-C DNA-Au/AgNCs.	1 fg/mL–1 ng/mL	0.14 fg/mL	Blood, milk, tap water	[135]
Electrochemistry (DPV)	Endotoxin	CTAB-CuNCs@Polymyxin B-Cu Foil	100 ag/mL–10 ng/mL	100 ag/ mL	Blood serum	[133]
Capillary biosensor	*S. typhimurium*	PAbs/BSA-FeNCs@MNPs	3.0 × 10^2^–3.0 × 10^6^ CFU/mL	14 CFU/mL	Spiked chicken samples	[48]

**Table 2:** Nanocluster-based biosensors for virus detection.

Biosensing Method	Analyte	Nanocluster	LR	LOD	Real Sample	Ref.
Fluorescence	HIV HBV HTLV-I	DNA-AgNCs	N/A	4.4 nM (HIV) 6.8 nM (HBV) 8.5 nM (HTLV-I)	N/A	[141]
	H5N1	DNA-AgNCs	500 pM–2 μM	500 pM	Fetal bovine serum	[142]
	Norovirus	DNA-AgNCs	20 nM–1.8 μM	18 nM	N/A	[143]
	HIV	DNA-AgNCs@GO	10 nM–100 nM	1.18 nM	Human serum	[144]
	HIV	DNA-AgNCs@exonuclease III	50 pM–5 nM	35 pM	Human serum	[145]
	HBV	DNA-AgNCs@SiO_2_ NP	N/A	0.65 nM	Human serum	[146]
	HPV-16	DNA-AgNCs	5–100 nM	2 nM	Human serum	[147]
	HIV-1 HIV-2	DNA-AgNCs	0.2–700 nM	11 pM	Human serum	[148]
	HIV H1N1 H5N1	DNA-AgNCs	5–2000 nM (HIV) 0–250 nM (H1N1) 50–500 nM (H5N1)	3.53 nM (HIV) 0.12 nM (H1N1) 3.95 nM (H5N1)	Fetal bovine serum	[149]
	HIV/HCV	AgNCs	10 fM–100 pM	1.4 fM	N/A	[150]
	HBV	PEI-AgNCs@MoS_2_ nanosheets	5–30 nM	5 nM	N/A	[151]
	HIV	DNA-AgNCs@ CNPs	1–50 nM	0.40 nM	N/A	[46]
	SARS-CoV-2	DNA-AgNCs	0.30–10.0 nM	0.30 nM	Clinical throat swab samples	[93]
	HBV	DNA-AgNCs@ CIDDC	1–20 nM	0.11 nM	Human serum	[152]
	HIV	DNA-AgNCs	15–150 nM	3.18 nM	N/A	[153]
	RSV	DNA-AgNCs@ MWCNTs	31.25 nM–2.00 μM	24.00 nM	N/A	[154]
	HIV HBV	DNA-AgNCs/GO	N/A	1 nM (HIV) 0.5 nM (HBV)	N/A	[155]
	HIV	Streptavidin-AuNCs	Up to 1000 pg/mL	5 pg/mL	Plasma	[139]
	SARS-CoV-2	BSA-AuNCs	N/A	20 pM	N/A	[140]
	SARS-CoV-2 Delta	DNA-CuNCs	0.5 pg/μL–50 ng/μL	5 fg/μL	N/A	[156]
	HBV	DNA-CuNCs@CRISPR-Cas12a enzymes	0.5–100 pM	0.54 pM	Human serum	[35]
	MPXV	DPA-CuNCs	N/A	0.096 nM (fluorescence) 0.114 nM (electrochemical)	Serum	[157]
	CTV	CP25 Pr-CuNCs	400 pg/mL–25 ng/mL	220 pg/mL	Fresh extract of petioles and vessels from young leaves of healthy and infected citrus plant	[32]
	HTLV-I	DNA-GQDs @CuNCs/exonuclease III	20 pM–12 nM	10 pM	Human serum	[158]
	HCV	3D-DNA Walking Nanomachine-CuNCs@CHA	100 pM–2 nM	42.4 pM	N/A	[159]
Colorimetry (creatinine, H_2_O_2_, and ABTS)	HBV	dsDNA@CuNCs	12 × 10^9^–12 × 10^13^	12 × 10^9^ molecules for analyzing DNA	Human serum	[136]
Colorimetry	ASFV	DNA-Magnetic NCs		19.8 pM	Serum, tissue, feed, and lymph nodes	[138]
Dual-mode photothermal/colorimetry	SARS-CoV-2 nucleocapsid protein	PEI@Fe_3_O_4_ NCs@Au nanoshells	100 pg/mL–1000 ng/mL	43.64 pg/mL	Clinical samples	[137]
Electrochemistry (potentiometry and amperometry)	SARS-CoV-2	DHLA-PdNCs		100 copies µL^−1^ of fragment N1	Nasopharyngeal exudate samples	[163]
Electrochemistry (DPV)	HIV	DNA-/G -AuNCs@exonuclease III	0.1 fM–100 nM	30 aM	Human serum	[160]
Electrochemiluminescence (ECL)	HPV	magnetic beads-AuNCs@MnO_2_ nanosheets	1.00 × 10^−16^–1.00 × 10^−8^ M	6.8 aM	Clinical cervical brush specimens	[161]
Electrochemiluminescence (ECL)	HPV	Met-AuNCs/SH-ssDNA-Fc@Cas12a	10^−12^–10^−8^ M	0.48 pM	Human blood	[162]

## Data Availability

Not applicable.
